# Chronosequence Resampling Elucidates Tree Community and Forest Structure Recovery Patterns in Restored Tropical Rainforest

**DOI:** 10.1002/ece3.72033

**Published:** 2025-08-24

**Authors:** Eveliina Korkiatupa, Geoffrey M. Malinga, Sille Holm, Wouter van Goor, Richard Kigenyi, Anu Valtonen

**Affiliations:** ^1^ Department of Environmental and Biological Sciences University of Eastern Finland Joensuu Finland; ^2^ Department of Biology Gulu University Gulu Uganda; ^3^ Department of Ecology Swedish University of Agricultural Sciences Uppsala Sweden; ^4^ Face the Future Wageningen the Netherlands; ^5^ Uganda Wildlife Authority Fort Portal Uganda; ^6^ Department of Ecology, Environment and Geoscience Umeå University Umeå Sweden

**Keywords:** Kibale National Park, seed dispersal, succession, tree planting, tropical reforestation, vegetation

## Abstract

Forest restoration is an essential tool for conserving biodiversity in tropical regions; yet, restoration outcomes in the Afrotropics remain largely understudied. Here, we investigated how the forest structure, tree diversity, community, life‐history traits and habitat associations recovered over three decades of active restoration in an East African rainforest in Uganda. The vegetation surveys were initially conducted in 2013 and repeated in 2021. Altogether, the study included 45 actively restored forest sites (aged 4–26 years) and 10 primary forest reference sites. The results showed increased tree taxa richness, basal area, tree height and community similarity (i.e., the similarity of community composition of restored forests to the composition of primary forest) along the age gradient. After 20 years of planting, Simpson's diversity and canopy cover reached similar values recorded in the reference primary forest. In contrast, restored forests had not attained levels of tree taxa richness, basal area, stem density, or community similarity comparable to those of the reference primary forest within three decades. We found an age gradient from younger restored to older restored to the primary forest in the composition of tree communities. The proportion of species with animal‐dispersed seeds was similar in the restored and the primary forest. The proportion of shade‐tolerant and forest‐interior species had increased along the age gradient in the 2021 survey. In conclusion, forest structure, diversity and community showed early signs of recovery, but the rate of change slowed over time.

## Graphical abstract

We studied forest structure, tree diversity, traits and community recovery patterns in an age gradient of actively restored Afrotropical rainforest in 2013 and 2021. The results showed early signs of recovery for tree taxa richness, basal area, stem density and community similarity with variable recovery rates.

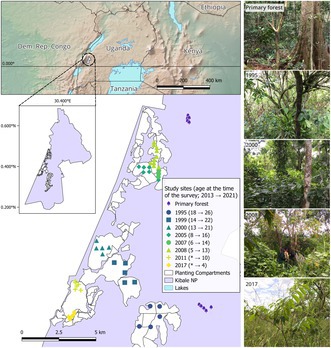

## 1 Introduction

Deforestation and forest degradation threaten biodiversity and essential ecosystem services provided by rainforests (IPBES 2019; Boulton et al. 2022). Vancutsem et al. (2021) estimated that 218.7 million ha of tropical moist forests were lost between 1990 and 2020. In some tropical regions, most of the forest cover has already gone; for example, Uganda has lost 92% of its historical forest cover (Aleman et al. 2017). In such regions, restoration is vital to conserve the remaining biodiversity and sustain ecosystem services that forests provide (Gann et al. 2019; Di Sacco et al. 2021).

Most studies describing the dynamics of tropical forest recovery have been conducted in the Neotropics (e.g., Guariguata and Ostertag 2001; Liebsch et al. 2008; Mesquita et al. 2015; Kulikowski et al. 2022; Maurent et al. 2023). Other regions, particularly Africa, remain comparatively understudied (Wainwright et al. 2018; Jakovac et al. 2021). Besides, tropical forest restoration research has focused on the natural recovery of vegetation after disturbances, with less emphasis on actively restored systems (Meli et al. 2017). Recent meta‐analyses have demonstrated that natural regeneration (i.e., passive restoration) facilitates faster vegetation recovery compared to active restoration (e.g., Crouzeilles et al. 2017). However, passive restoration studies, utilising natural regeneration, are often done under favourable conditions for tree regeneration, generating faster recovery than in active restoration (Reid et al. 2018). Nevertheless, in some areas, natural recovery is unattainable. Succession can be arrested, for instance, by frequent fires or a dense grass and shrub cover (Duncan and Duncan 2000; Duclos et al. 2013; Chazdon 2014; Wheeler et al. 2016). In these areas, the forest may recover only by implementing active strategies such as tree planting (Lamb et al. 2005; Chazdon 2014).

Succession drives ecosystem recovery during forest restoration. Tropical forest succession involves three phases based on gradual changes in vegetation structure, species composition and functional groups (Chazdon 2008; Suding and Hobbs 2009; Ghazoul and Sheil 2010; Elliott et al. 2013). The stand initiation phase is dominated by light‐demanding pioneer species for the first 10 years. Over the next 10–20 years, tree height and cover increase, leading to the recruitment of more shade‐tolerant species in the stem exclusion phase. Finally, in the understorey reinitiation phase, late‐successional species and several vegetation layers gradually establish themselves. This phase may take 100–200 years (Chazdon 2014; Ghazoul and Sheil 2010).

Several biotic and abiotic factors, along with land‐use history and intensity, may modify the trajectories and speed whereby the forest structure, diversity and community composition shift during succession (Jakovac et al. 2021; Maurent et al. 2023). Key abiotic factors altering successional trajectories include topography, soil type, microclimate and fire (Jakovac et al. 2021; Rochimi et al. 2021). Biotic factors encompass herbivory, the abundance of seed dispersers and predators, as well as competition between trees and other vegetation (Struhsaker 1997; Lawes and Chapman 2006; Duclos et al. 2013; Mantoani and Torezan 2016; Omeja et al. 2016; Piiroinen et al. 2017).

Whether active or passive restoration is used, the slow arrival of recruits (seed dispersal limitation) and/or poor germination, survival, or growth of seedlings (tree establishment limitation) can impede succession (Duncan and Chapman 1999; Flores and Holmgren 2021; Joyce et al. 2024). Seed dispersal limitation is often a stronger barrier to forest succession, especially for animal‐dispersed, large‐seeded and late‐successional species (Reid et al. 2015; Holl et al. 2017; Sangsupan et al. 2018; Peña‐Domene et al. 2018). Seed dispersal limitations can result from long distances to source populations (e.g., conservation areas, forest fragments, remnant trees), surrounding land use, life‐history traits of recruits (e.g., dispersal type, seed or fruit size), as well as the presence and diversity of seed dispersers (review by Jakovac et al. 2021). Again, this topic is most explored in Central and South America (e.g., van Breugel et al. 2019; Peña‐Domene et al. 2018; Zahawi et al. 2021; Rodrigues et al. 2019; Camargo et al. 2020), with limited research in the Afrotropics (Ssekuubwa et al. 2021; Abiem et al. 2023).

Here we investigate tree community and forest structure changes in an actively restored Afrotropical rainforest of Kibale National Park, Uganda. We focus on areas where natural succession was arrested and the forest recovery started only with reforestation (Duncan and Duncan 2000; Wheeler et al. 2016). In the studied large‐scale restoration area (10,000 ha), active restoration was initiated in 1995 (UWA‐FACE 2015). Our study represents a chronosequence resampling, with forest ages spanning from 4 to 26 years. The first survey was implemented in 2013; the second in 2021. We classified the study sites into four forest age groups: (1) younger restored forest (aged 4–10 years in 2021), (2) intermediate‐aged restored forest (aged 5–8 years in 2013, 13–16 years in 2021), (3) older restored forests (aged 13–18 years in 2013, 21–26 years in 2021) and (4) primary forest study sites.

We asked (Question 1; Q1) whether tree diversity, or variables describing the forest structure (basal area, stem density, canopy cover, tree height), differs among younger restored, intermediate‐aged restored, older restored and primary forests? We hypothesised that tree diversity, basal area, stem density, canopy cover and tree height would increase with the forest age as active restoration initiates secondary succession. As planted trees age and grow larger, the changing microhabitat attracts more seed dispersers, and the germination and growth of recruited seedlings induce higher species diversity and structural complexity (Elliott et al. 2022).

Secondly (Q2), do the proportions of species presenting different life‐history traits (seed dispersal types, seedling establishment guilds) and habitat associations differ among the forest age groups? If seed dispersal limitation is a common phenomenon in our study area, the proportion of animal‐dispersed species should be lower in the younger restored forests compared to the primary forest (Reid et al. 2015; Peña‐Domene et al. 2018). Shade‐tolerant and forest‐interior species should be more common in primary forests, while proportions of pioneer and forest non‐dependent species (occurring both in open and forest habitats) should be higher in younger restored forests.

Thirdly (Q3), we examined whether the tree community composition changes over the 8 years of recovery (between the surveys) and how much of the variation in compositions can be explained by forest age, distance to primary forest and elevation. We predicted that the similarity in community composition of restored forests to the primary forest composition would increase over the 8 years between the surveys; yet, the rate of change may differ among forest age groups. We expected compositional similarity to increase because planting should enhance growth conditions, and seed dispersal from the primary forest should bring new species and replace pioneers with later‐successional species (Elliott et al. 2013; Chazdon 2014). Finally, we investigated which abiotic and biotic environmental variables correlate with the observed community composition.

## 2 Materials and Methods

### 2.1 Study Area

Our study area was located in the Kibale National Park, Uganda, which represents mainly medium‐altitude Afrotropical moist forest with an area of 795 km^2^ (Struhsaker 1997; Hartter et al. 2011). The elevational gradient in the park varies between 900 and 1590 m above sea level, with higher elevation in the north compared to the south. Consequently, the vegetation changes from medium‐altitude tropical moist forests in the north and east to savannahs in the south and west (Omeja et al. 2011; UWA‐FACE 2015). The rainy season occurs twice yearly: from March to May and from August to November. The mean annual precipitation was 1646 mm, and the mean annual monthly temperature ranged between 16°C_min_ and 28°C_max_ (data between 1970 and 2020; Chapman et al. 2021).

Evergreen and semi‐deciduous primary forest covers most of the national park (UWA‐FACE 2015). At least 351 tree species are found in Kibale, with species composition varying along the elevational gradient (Wing and Buss 1970; Howard et al. 1996). The topography is undulating; hence, vegetation composition varies between the valley bottoms and hills (Struhsaker 1997). Common species include *Uvariopsis congensis*, *Celtis* spp., *Pterygota mildbraedii*, *Markhamia lutea* , *Olea welwitchii*, *Gambeya* spp., *Pseudopsondias microcarpa* and *Cynometra alexandri*, among others (Howard et al. 1996; UWA‐FACE 2015). The forest canopy can reach a height of over 30 m (Wing and Buss 1970), with some tree species capable of growing to 45 m (Kalema and Hamilton 2020). The total basal area in the primary forest can exceed 35 m^2^/ha (Skorupa 1988).

A mosaic of different vegetation types can be found within Kibale, including closed‐canopy primary forests, grasslands, swamps and successional forests (Wing and Buss 1970; Struhsaker 1997; UWA‐FACE 2015). In the successional forests, the cover of understorey vegetation changes along successive phases. During the initial phase, grasses, especially *Cenchurus purpureus* (elephant grass), are dominant but are replaced by different woody shrubs and herbaceous perennials, including *Acanthus pubescens*, which proliferate under the pioneer trees (Wing and Buss 1970). During subsequent phases, the canopy cover of young forests is sufficiently sparse for light to reach the forest floor, where herbaceous, shade‐tolerant plants, such as *Marantochloa* spp., supplant most grasses and shrubs. In later phases, the canopy closes, eventually resulting in very little or no herbaceous understorey.

**FIGURE 1:**
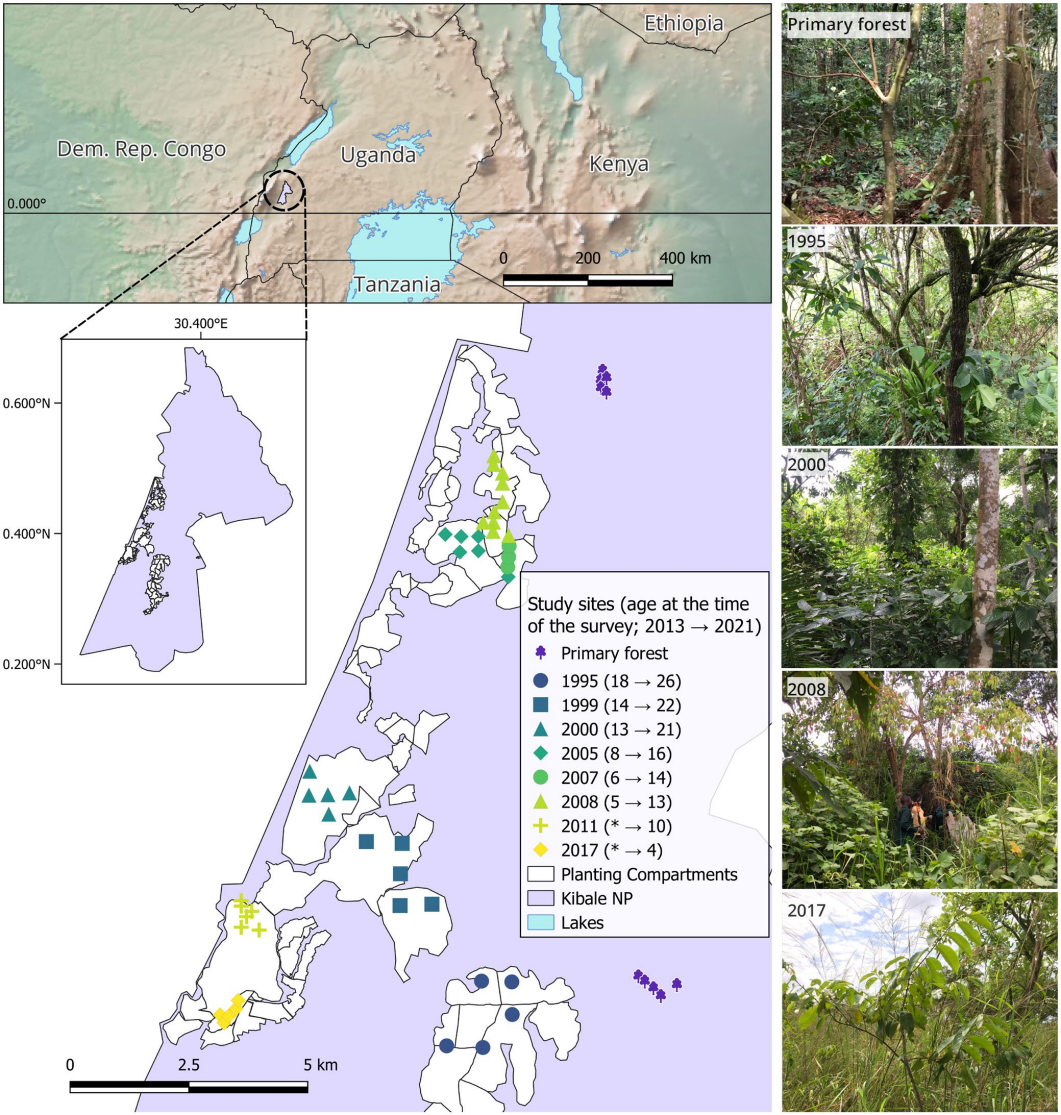
Map of restoration project area and vegetation survey study sites in Kibale National Park, Uganda. The first survey took place in 2013 and the second in 2021; in the legend, an asterisk indicates that the study site was not surveyed in 2013. Map layers: Waterbodies: Africa—Waterbodies dataset (World Bank 2018, CC‐BY 4.0); country borders, rivers, lakes at the continent level, shaded relief with elevation: Natural Earth dataset (Natural Earth 2023; Public Domain). The map was created in QGIS Desktop 3.20.3 (QGIS 2021). Photos: A. Valtonen.

This study was conducted in a forest restoration project area and adjacent primary forest reference sites in Kibale National Park (figure 1; Omeja et al. 2011; UWA‐FACE 2015; Wheeler et al. 2016). Between the 1970s and 1990s approximately 10,000–15,000 ha of the southern part of the park lost its forest cover due to illegal human activity, specifically, land clearance for agriculture; for a more detailed history, refer to Struhsaker (1997), Chapman and Lambert (2000), and UWA‐FACE (2015). Of this area, 2600 ha was found to be suitable for natural regeneration, being enclosed by existing forest (Riemer 2020). Natural regeneration areas were actively protected from fire through patrolling and the establishment of firebreaks. In other parts, the land transitioned permanently into grass‐ and shrubland, where tree regeneration was virtually absent (UWA‐FACE 2015; Wheeler et al. 2016). In 1994, the Uganda Wildlife Authority (UWA) and Face the Future (FACE) launched a reforestation project. Since 1995, active restoration has taken place nearly annually via planting trees, removing grass around seedlings, preventing fires and removing invasive species such as *Lantana camara* . The project had planted over 4400 ha with local tree species by 2023 (Riemer 2020; van den Tweel 2023). Our study sites were located in these actively restored areas (Figure 1).

The planted tree species included a mixture of pioneer species such as *Bridelia micrantha*, *Spathodea campanulata* and *Shirakiopsis elliptica*; “intermediate successional” species such as *Albizia gummifera* , *Croton macrostachys, Cordia africana*, *Warburgia ugandensis*, *Mimusops bagshawei*, and *Prunus africana* and climax species such as *Gambeya albida* and *U*. *congensis* (Omeja et al. 2011; UWA‐FACE 2015; Wheeler et al. 2016; Riemer 2020). During the first years of the project, almost 40 tree species were planted in the area (Wheeler et al. 2016). The species composition of planted trees has changed during the later years, with the number of planted species reduced to around 10, comprising only those with a high seedling survival rate. The trees were planted in a 5 × 5 m grid.

### 2.2 Vegetation Surveys

In this study, we utilised vegetation survey data collected in 2013 (Nyafwono et al. 2014). The data covered 34 study sites representing six different‐aged, actively restored forests (planted between 1995 and 2008), as well as 10 primary forest sites (Figure 1). In 2021, we repeated the survey at the same study sites using the same survey protocol while appending 11 additional study sites across actively restored compartments planted in 2011 and 2017 (Figure 1). Distances between the study sites varied between 110 m and 1914 m within the sites planted in the same year and between 110 m and 15,783 m among all study sites.

At each study site, the vegetation survey was conducted following a nested plot design, wherein all plots shared one corner (Appendix S1; Figure S1). Large trees (diameter at breast height (DBH) ≥ 20 cm) were recorded in a 20 × 40 m plot, medium trees (DBH ≥ 10 and < 20 cm) in a 20 × 20 m plot, small trees (DBH ≥ 5 and < 10 cm) in a 20 × 10 m plot and saplings (DBH < 5 cm; including seedlings) in a 10 × 10 m plot. Unfortunately, we were unable to locate the plots exactly in the same position in 2021 as in 2013. This was because, for the plots surveyed in 2013, we had information only on the coordinates of the shared corner of the nested plots, but the orientation of the plot from that corner was unrecorded. To maximise the shared area in the two survey times, in the 2021 survey, we placed the centre of the plot at the known coordinates from the 2013 survey (Appendix S1; Figure S1).

Each tree was identified at the species level, or, if assignment to species was impossible, at the genus level, or listed as unidentified. Unidentified taxa represented only 1% of counted stems (see Appendix S1 for more information). In the field, all recorded trees were further classified as “planted” or “not planted” based on their location in the planting grid. Most of the trees in the planting grid were pre‐grown seedlings, but all the remnant trees or naturally regenerating seedlings were left on the site during the restoration planting. Therefore, in our vegetation survey, the stems located on the grid were classified as “planted” even though a few of these trees might have been naturally sprouted. In unclear cases or if the stem was located off the grid, it was classified as “not planted”.

For each plot, we calculated the following variables describing the vegetation diversity and structure: (1) tree taxa richness (number of tree taxa in 20 × 40 m plot), (2) Simpson diversity index, (3) the total estimated basal area (m^2^/ha, for trees DBH ≥ 5 cm) and (4) the total estimated stem density (stems/ha). The tree taxa richness and Simpson diversity index were calculated using estimated stem densities (stems/ha) of the whole tree community, i.e., summing all tree size classes. Simpson's diversity index (*D*) was calculated as in Clarke and Gorley (2006): 1 − *D* = 1 − Σ_*i*_ [*N*_*i*_ (*N*_*i*_ − l)]/[*N*(*N* − 1)], where *N*_*i*_ = number of individuals in species *i*, and *N* = total number of individuals (includes both species richness and evenness; Magurran and McGill 2011).

In the 2021 vegetation survey, we further recorded the following variables from the 20 × 40 m plots: (5) mean tree canopy cover (%), and (6) mean tree height (m). The tree canopy cover was measured with the phone application *CanopyCapture* (Patel 2018) from the corners and in the middle of the 20 × 40 m plot, i.e., including five measurements. Tree height was measured only in the restored study sites; whereas, for the primary forest, we could only use a literature‐based height of 30 m (Wing and Buss 1970). See more detailed information on measuring the basal area, canopy cover and tree height in Appendix S1. As biotic environmental variables, we estimated the following understorey vegetation covers (%) of (7) *Cenchurus purpureus*, (8) *Acanthus pubescens* , (9) *Lantana camara* , and (10) *Marantochloa* spp. (see details in Appendix S2). (11) Elevation (m above sea level) for each study site was extracted from a digital elevation model (Regional Centre for Mapping of Resources for Development 2018) in QGIS (2021).

For the statistical analyses, we divided the study sites into four forest age groups: (1) younger restored forest (planted in 2011 and 2017), (2) intermediate‐aged restored forest (planted in 2005, 2007 and 2008), (3) older restored forest (planted in 1995, 1999 and 2000), and (4) primary forest study sites (Figure 1). These groups approximated the successional phases: younger restored forests were in the stand initiation phase, intermediate‐aged restored forests were transitioning from stand initiation to the stem exclusion phase, and older restored forests were transitioning to the understorey reinitiation phase.

### 2.3 Data Analyses

We fitted linear mixed models (LMM) in SPSS (version 19.0.2.0 IBM Corp 2023) to determine if the three forest age groups (intermediate‐aged restored, older restored and primary forest), or the two survey times (2013 vs. 2021), differed in variables describing their tree diversity or forest structure (Q1). Younger restored forests were excluded from these analyses because they were measured only in the 2021 survey. In these models, the response variables included (1) tree taxa richness, (2) Simpson's diversity index, (3) estimated total basal area (m^2^/ha), (4) estimated total stem density (stems/ha), (5) mean canopy cover (%) and (6) mean tree height (m). The survey time and forest age group (and their interaction) were designated as fixed variables. For the models of canopy cover and tree height, only the forest age group was used as a fixed variable, as this data was collected solely in 2021. Additionally, the study site (nested in planting year) was set as a random variable in all models except canopy cover and tree height models (see model details in Appendix S1). If significant differences were found, we performed pairwise tests (LSD) to ascertain which forest age groups differed from each other (Appendix S1). The primary forest age group in the LMM model for comparing tree height was excluded, as it was not recorded in the field (see above). Instead, we used a one‐sample t‐test to ascertain whether the tree heights of restored forests (intermediate‐aged and older restored) differ from the literature‐based primary forest tree height (30 m; Wing and Buss 1970).

To inspect differences in tree taxa richness (Q1), we also produced individual‐based species accumulation curves using the iNEXT package in R (Hsieh et al. 2016; R Core Team 2023). Rarefaction and extrapolation curves were generated for each forest age group and both survey times.

We also estimated the growth rate of planted trees based on their DBH. The detailed information of the methods and results for planted tree growth is presented in Appendix S2.

We ran Fisher's exact tests in R to examine whether the proportions of species with different traits differed among the forest age groups (Q2; see Appendix S1). The studied traits included dispersal type (animal vs. non‐animal), seedling establishment guild (shade bearer [here shade‐tolerant], non‐pioneer light demander, pioneer, or swamp; classification as in Hawthorne 1995) or habitat association type (forest interior, forest edge, forest generalist, forest non‐dependent, riverine forest, woodland; classification as in Howard et al. 1996). Trait information was collected from literature (for sources, see Appendix S1: Table S1).

We used multivariate analyses to visualise the community compositions and to unravel whether the similarity of community compositions between the restored and primary forests had increased over the 8 years of restoration (Q3). In all multivariate statistics, we applied the presence–absence transformation, i.e., we used the Sørensen index for the community data. We chose this transformation to lessen the effect of slight differences in study plot locations between the two survey times. All multivariate analyses were conducted in PRIMER‐E 6.1.15 and the PERMANOVA+ add‐in (Clarke and Gorley 2006; Anderson et al. 2008).

We used Principal Coordinates Analysis (PCO; Anderson et al. 2008) to visualise tree community changes (Q3). PCO is an ordination method that projects samples onto axes based on their inter‐point dissimilarities in Euclidean space. We chose PCO because a more commonly used NMDS yielded “degenerate” solutions with our data (Anderson et al. 2008: 121–122). The degenerate solution means that NMDS collapses if there are a few samples (in our case study sites) with almost no species. In our case, NMDS produced figures where most samples aggregated on top of each other, while one to two outliers (i.e., study sites with low species richness) were separated. In this case, PCO provides a solution without having to remove “outliers” that still provide ecologically important information. We generated PCOs for the whole tree community, with all size classes and separately for the four size classes (saplings, small, medium and large trees), including both survey times. To explore correlations between the measured environmental variables and the ordination axes, we used community composition data from 2021, when all environmental variables were measured. The abiotic and biotic environmental variables included were (1) forest age, (2) latitude, (3) longitude, (4) elevation, (5) distance to primary forest, (6) canopy cover and the four (7–10) understorey vegetation cover estimates listed above (see also Appendix S2 for the vegetation covers).

We ran paired t‐tests to determine if the compositional similarity of restored forests to primary forests had changed between the two surveys (Q3), using R. First, we calculated the mean compositional similarity (Sørensen similarity index) between each restored forest study site and all of the primary forest study sites, separately for both survey times. Second, we performed paired *t*‐tests to assess if compositional similarity to the primary forest changed between 2013 and 2021. Only the intermediate‐aged and older restored forests were included, as the younger restored forests were surveyed only in 2021. We also tested, using the paired *t*‐test, if the compositional similarity among the primary forest study sites had changed (mean compositional similarity to all other primary forest sites).

Finally, we performed two distance‐based linear models (DistLM; Anderson et al. 2008). These were fitted per survey time to discover how large a proportion of variance in tree community composition was explained by (1) age, (2) the study site's distance to the primary forest and (3) elevation (Q3). We also included the (4) latitudinal and (5) longitudinal coordinates of the study site in the models to account for their spatial configuration. We fitted DistLM models, first using the entire dataset and second, excluding the planted tree individuals from the dataset. We did this to ensure that the gradients observed in the tree community compositions were not generated by the differences in the compositions of the planted trees across different years (see Appendix S1).

## 3 Results

### 3.1 Recorded Tree Taxa

The 2013 and 2021 data covered 89 tree taxa identified to a species or genus level (Appendix S1; Table S1). Of the identified taxa, 60 were recorded in the restored forests, 67 in the primary forests, and 38 in both (Appendix S1; Figure S2). The 2013 survey found 79 tree taxa, while the 2021 survey found 73 taxa. The most common taxa in restored forests were *Bridelia micrantha*, *Funtumia* sp., *Shirakiopsis elliptica* and *Albizia grandibracteata* (based on their estimated stem density/ha; Appendix S1: Table S2). Correspondingly, the most common taxa in primary forests were *Rinorea* sp., *Lovoa swynnertonii*, *Uvariopsis congensis* and *Monodora myristica (*Appendix S1: Table S2).

### 3.2 Diversity and Vegetation Structure (Q1)

**FIGURE 2:**
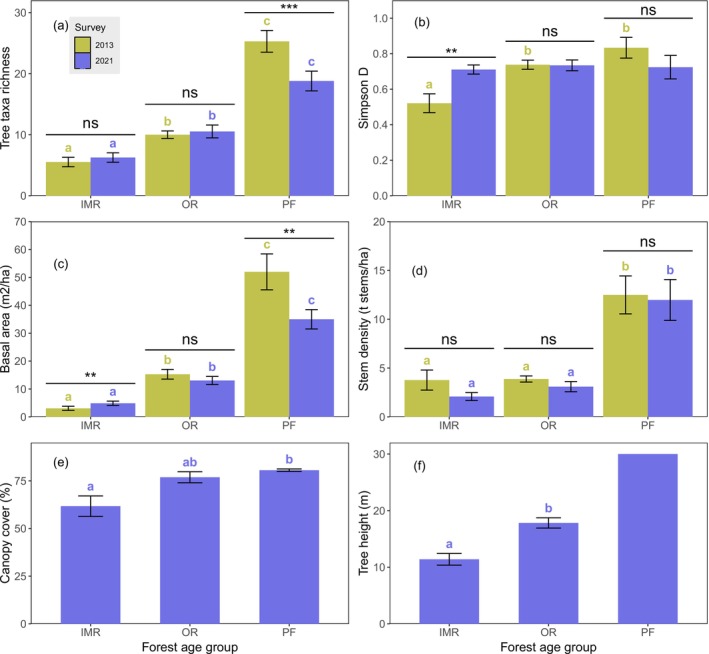
Bar plots presenting the mean and standard error of (a) tree taxa richness, (b) Simpson's diversity, (c) total estimated basal area (m^2^/ha), (d) estimated stem density (thousand stems/ha), (e) canopy cover (%) and tree height (m) for each forest age group. LSD post hoc test results are shown in the panels (the corresponding linear mixed model test results are presented in Tables 1 and 2). Different letters indicate statistically significant differences among forest age groups (*p* < 0.05); colours represent comparisons within each survey year. Statistical differences between the two survey years are presented as *p* > 0.05 (ns), *p* < 0.01 (**), *p* < 0.001 (***). The primary forest age group was discarded from the analyses for tree height (since it was not measured at our primary forest study sites; the mean tree height for the primary forest was derived from Wing and Buss (1970) and used in panel f). Forest age groups: IMR = intermediate‐aged restored (aged 5–8 years in 2013, 13–16 years in 2021), OR = older restored (aged 13–18 years in 2013, 21–26 years in 2021) and PF = primary forest.

The tree taxa richness, Simpson diversity, total basal area, stem density, mean canopy cover and mean tree height increased significantly from intermediate‐aged restored forests towards primary forests (LMMs; Table 1; Table 2; Figure 2; Appendix S1: Figure S3). However, a significant interaction between survey time and age group was found in tree taxa richness, Simpson's diversity and basal area, but not in stem density. Therefore, while we found a general increase in taxa richness and basal area between the two study times, at the same time interval, these variables showed a decreasing pattern in the primary forest (LSD tests; Figure 2a,c; Table 1). Simpson's diversity, on the other hand, showed an increase only in intermediate‐aged restored forests (LSD tests; Figure 2b; Table 1). Based on pairwise LSD tests, the restored forests had lower tree taxa richness, basal area and stem density than primary forests at both survey times (Figure 2a,c,d). Simpson's diversity (in 2013) and canopy cover (in 2021) were lower only in the intermediate‐aged restored forests versus primary forests (Figure 2b,e). The tree height of the restored forests was significantly lower compared to the literature‐based tree height of primary forests (*t*(43) = −10, *p* < 0.001; one‐sample *t*‐test). Furthermore, there was plenty of variation among study sites in all diversity and forest structure variables when plotted separately for each forest age and planting year (Appendix S1; Figure S3). The corresponding changes in the cover estimates of the understorey vegetation and growth of planted trees along the chronosequence are presented in Appendix S2 (the text and Figures S1–S2).

**TABLE 1:** Linear mixed model (LMM) results for: (a) tree taxa richness, (b) Simpson's diversity, (c) basal area (m^2^/ha) and (d) stem density.

Response variable	Source	Numerator df	Denominator df	*F*	Sig.
(a) Tree taxa richness	**Intercept**	**1**	**41.0**	**505.6**	**< 0.001**
	**Survey time**	**1**	**41.0**	**16.2**	**< 0.001**
	**Age group**	**2**	**41.0**	**65.5**	**< 0.001**
	**Survey time × age group**	**2**	**41.0**	**25.6**	**< 0.001**
(b) Simpson's diversity	**Intercept**	**1**	**41.0**	**1193.7**	**< 0.001**
	Survey time	1	41.0	0.7	0.409
	**Age group**	**2**	**41.0**	**6.2**	**0.004**
	**Survey time × age group**	**2**	**41.0**	**8.6**	**< 0.001**
(c) Basal area (m^2^/ha)	**Intercept**	**1**	**41.0**	**360.0**	**< 0.001**
	**Survey time**	**1**	**41.0**	**12.2**	**0.001**
	**Age group**	**2**	**41.0**	**107.6**	**< 0.001**
	**Survey time × age group**	**2**	**41.0**	**10.4**	**< 0.001**
(d) Stem density (stems/ha)	**Intercept**	**1**	**41.0**	**171.5**	**< 0.001**
	Survey time	1	41.0	1.8	0.190
	**Age group**	**2**	**41.0**	**34.4**	**< 0.001**
	Survey time **×** age group	2	41.0	0.2	0.782

*Note:* In each model, fixed effects (Type III) included survey time, forest age group, and their interaction. Statistically significant results are highlighted in bold. Post hoc test (LSD) results are shown in Figure 2.

**TABLE 2:** Linear mixed model (LMM) results for (a) mean canopy cover (%), and (b) mean tree height (m).

Response variable	Source	Numerator df	Denominator df	*F*	Sig.
(a) Canopy cover (%)	**Intercept**	**1**	**41**	**772.166**	**< 0.001**
	**Age group**	**2**	**41**	**5.421**	**0.008**
(b) Tree height (m)	**Intercept**	**1**	**32**	**426.464**	**< 0.001**
	**Age group**	**1**	**32**	**20.596**	**< 0.001**

*Note:* In each model, the fixed effect included only the forest age group. The tree height could be compared only among intermediate‐aged and older restored forests (see text for more details). Statistically significant results are highlighted in bold. Post hoc test (LSD) results are shown in Figure 2.

**FIGURE 3:**
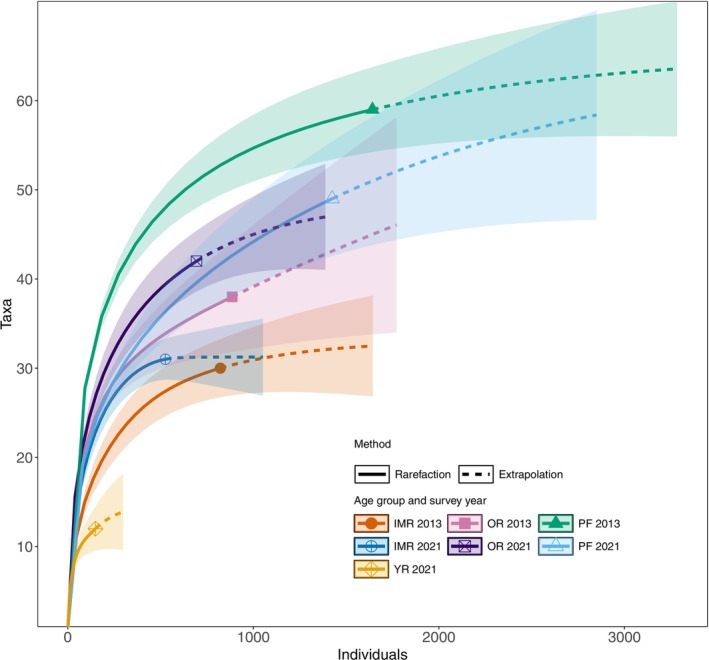
Individual‐based species accumulation curves for the forest age groups during the survey years 2013 and 2021. YRs were surveyed only in 2021. Graphs were generated with the iNEXT package (Hsieh et al. 2016) in R (R Core Team 2023). 95% confidence intervals are shown in lighter colors. Forest age groups: YR = younger restored (aged 4–10 years in 2021), IMR = intermediate‐aged restored (aged 5–8 years in 2013, 13–16 years in 2021), OR = older restored (aged 13–18 years in 2013, 21–26 years in 2021) and PF = primary forest.

Individual‐based species accumulation curves indicated that the taxa richness was significantly lower (i.e., 95% confidence intervals did not overlap) for younger and intermediate‐aged restored forests compared to primary forests (Figure 3). The older restored forests, however, had reached the primary forest taxa richness in 2021.

### 3.3 Differences in the Frequencies of Life‐History Traits and Habitat Associations (Q2)

**FIGURE 4:**
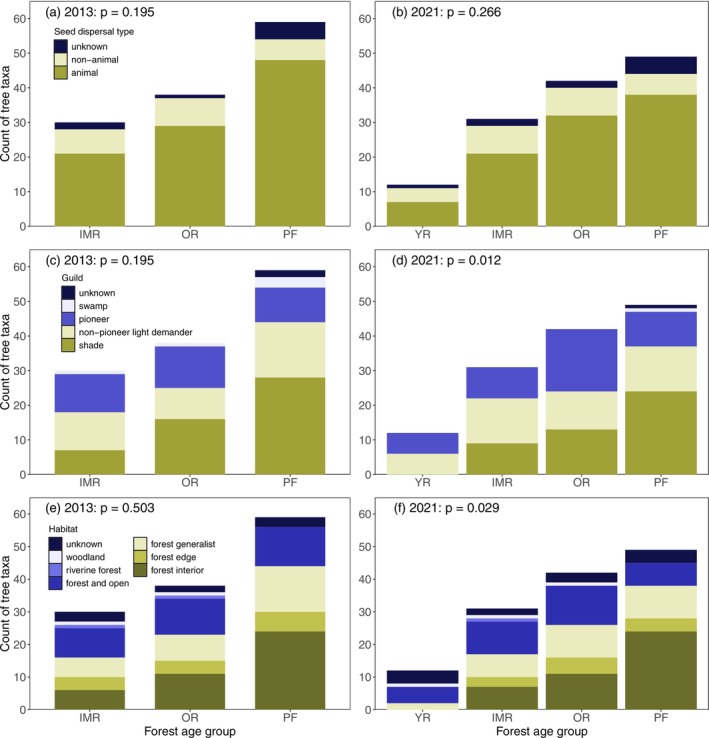
Seed dispersal type (a, b), germination and seedling establishment guild (c, d) and habitat association type (e, f) of tree taxa recorded in the forest age groups during the survey periods 2013 and 2021. YRs were surveyed only in 2021. Fisher's exact test significance results are presented for each trait and survey year (unknowns excluded from the tests). Forest age groups: YR = younger restored (aged 4–10 years in 2021), IMR = intermediate‐aged restored (aged 5–8 years in 2013, 13–16 years in 2021), OR = older restored (aged 13–18 years in 2013, 21–26 years in 2021) and PF = primary forest.

The majority of tree taxa across all age groups represented animal‐dispersed species, and proportions of different seed dispersal types did not differ significantly among forest age groups (Figure 4a,b). The proportions of shade‐tolerant species increased towards older forests, but the difference was statistically significant only in 2021 (Figure 4c,d). In 2021, no shade‐tolerant species were detected in the younger restored forests, while their proportion was approximately 30% in the intermediate‐aged and older restored forests and 50% in the primary forest study sites. Furthermore, the proportion of forest‐interior species increased along the age gradient, although the difference in frequencies among categories was significant only in 2021 (Figure 4e,f). In 2021, restored forests had more forest‐non‐dependent species (forest and open class) compared to primary forests, whereas primary forests had more forest‐interior species (Figure 4e,f). Examples of animal‐dispersed, shade‐tolerant, and forest‐interior taxa that had spread to the restored forests included *Allophyllus dummeri*, *Cassipourea ruwensoris*, *Gambeya* sp., *Mimusops bagshawei*, *Noronhia africana*, *Rothmania* sp., *Tabernaemontana* sp. and *Uvariopsis congensis* (Appendix S1: Table S1). Out of these, *Gambeya* sp., *M. bagshawei* and *U. congensis* could be either the progeny of planted trees or dispersed from the primary forest, while others represented naturally dispersed and established trees (Appendix S1: Table S1).

### 3.4 Community Compositions (Q3)

**FIGURE 5:**
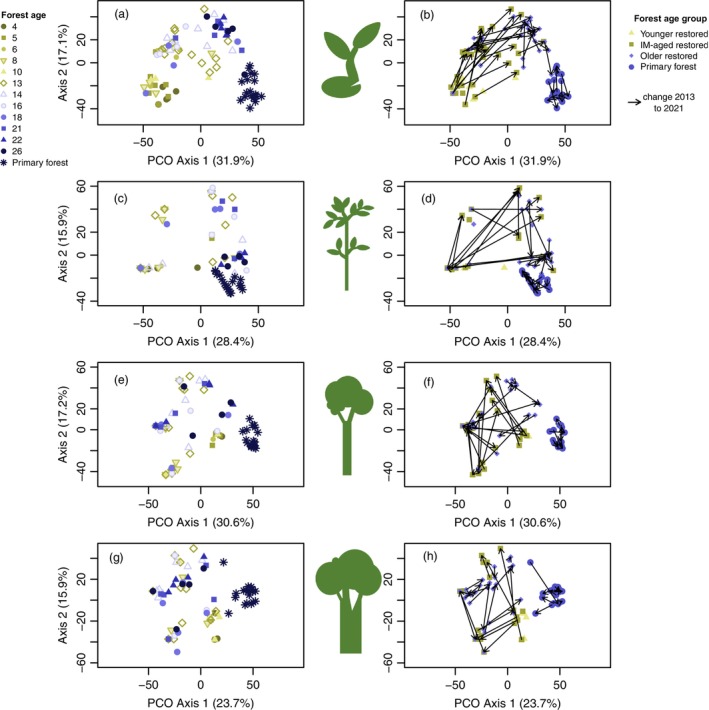
PCO ordinations of communities of saplings (a, b), small trees (c, d), medium trees (e, f) and large trees (g, h) (based on presence/absence transformation, i.e., the Sørensen similarity index). On the left side, the symbols show forest age (a, c, e, g) and on the right side, the changes within each study site between the years 2013 and 2021 (b, d, f, h). Percentages show how much the axis is explaining the total variation. Study sites that had no stems in the specific size class were excluded. IM‐aged intermediate‐aged.

Based on PCO ordinations, the tree community compositions of restored forests were largely distinct from primary forests both when all size classes were combined (Appendix S1; Figure S4) and when size classes were analysed separately (Figure 5). The sapling and small tree communities in some of the oldest restored study sites started to approach the community compositions found in primary forest reference sites (Figure 5a–d). However, communities of medium and large trees along the restoration gradient remained distinct from corresponding primary forest communities (Figure 5e–h). Moreover, the direction of community change formed a clear pattern in sapling and small tree communities; younger and intermediate‐aged restored communities moved systematically towards older restored communities, while older restored communities moved only very slightly towards primary forest communities (Figure 5b,d). For medium and large trees, the directional patterns were indistinct (Figure 5f,h).

Overall, the communities of the restored forests became slightly more similar to the primary forest in the 8 years (paired *t*‐test: *t* (33) = −4.14, *p* < 0.001; mean similarity 9.5% in 2013 and 13.2% in 2021; Appendix S1; Figure S5). Within forest age classes, community similarity to primary forest increased in intermediate‐aged restored forests (paired *t*‐test: *t* (18) = −4.94, *p* < 0.001, mean similarity 4.7% in 2013 and 9.8% in 2021), whereas no significant change was evident in older restored forests (paired *t*‐test: *t* (14) = −1.3, *p* = 0.2137; mean similarity 15.7% in 2013 and 17.6% in 2021) over the 8 years. There was no change in community similarity among primary forest study sites (paired *t*‐test: *t* (9) = 1.24, *p* = 0.247; mean similarity 55.6% in 2013 and 53.6% in 2021).

### 3.5 Environmental Variables Explaining the Tree Community Compositions (Q3)

In all PCO ordinations, the horizontal Axis 1 correlated most strongly with forest age (Appendix S1; Figure S6). Multiple environmental variables, including elevation and latitude, correlated with PCO Axis 2, which separated the younger restored forest into one group and intermediate‐aged and older restored forests into a second group (Appendix S1; Figure S6).

Based on the DistLM models, age explained the largest proportion of variation (25% to 36%) in the tree community compositions (Appendix S1; Table S3). Also, distance to primary forest, elevation, and latitude and longitude of the study site were statistically significant in explaining the tree community compositions (Appendix S1; Table S3). The DistLM model results remained qualitatively similar even after the planted tree individuals were removed from the dataset (Appendix S1; Table S4).

## 4 Discussion

Our relatively long chronosequence study illuminates the recovery patterns of an actively restored rainforest in the Afrotropics, emphasising that the different structural and diversity characteristics change at different rates along the restoration chronosequence gradient. As expected, the forest structure, tree diversity and community similarity increased with the forest age. However, most characteristics describing forest structure, diversity and community (e.g., basal area, stem density and community composition) of the restored forests were still considerably different from those of the primary forest. Our results are in line with previous studies conducted in Kibale (Omeja et al. 2011; Wheeler et al. 2016; Ssekuubwa et al. 2022), as well as the Neotropics, West Africa and Australia, reporting different recovery times for different variables describing the forest structure, diversity and function (e.g., Liebsch et al. 2008; Shoo et al. 2016; Poorter et al. 2021).

Our hypothesis that the proportion of animal‐dispersed species would increase from younger restored forests to primary forests was incorrect, as the proportions were equal in all forest age groups. Only a few earlier restoration studies have compared proportions of seed dispersal type along an age gradient, thereby hindering direct comparison of the results. However, one study showed that the proportion of non‐animal‐dispersed species decreased as a function of forest age in a long‐term passive restoration study in Panama (Estrada‐Villegas et al. 2022). Also, Peña‐Domene et al. (2018) showed that dispersal limitation of animal‐dispersed species would be higher in pastures than in restoration forests. Well in line with successional theory (Elliott et al. 2013; Chazdon 2014), the proportions of shade‐tolerant and forest‐interior species increased and forest non‐dependent species (occurring both in open and forest habitats) decreased along the age gradient towards primary forest (in the 2021 survey).

### 4.1 Structural Recovery at the Beginning of Secondary Succession

In just two decades, the canopy cover of the restored forests recovered, as the first structural component, to similar levels as found in the primary forest reference sites. This will probably support the recovery process of other forest attributes, as the lack of canopy cover is one of the critical environmental filters inhibiting secondary succession in tropical rainforests (Werden et al. 2020; Lindell et al. 2013; Elliott et al. 2022). The closing canopy, resulting from the growth of planted trees, will likely be an important factor facilitating the recovery, as the expanding canopy cover limits light availability, shades out grasses and thus facilitates tree establishment, growth and survival (Hooper et al. 2002; Sady et al. 2010; Osuri et al. 2022; Elliott et al. 2022; Ferreira and Vieira 2024). Furthermore, diminishing light conditions enable species turnover (Swaine and Whitmore 1988; Elliott et al. 2022), which then slowly modifies the community composition of trees.

Even though the canopy cover formed in a relatively short time, the tree height and basal area of restored forests are still significantly lower compared to the primary forest. This emphasises that the large trees (here DBH ≥ 20 cm) still exist in fewer numbers in the restored forests after 20–26 years of restoration planting compared to primary forest. Large (and old) trees form most of the total tree biomass, store considerable amounts of carbon and have an important role in forest architecture (Lindenmayer et al. 2012; Lutz et al. 2018). Large trees provide food and habitats for other species to grow and nest, as well as affect the growth conditions of other vegetation by modifying microclimate, hydrology, light and nutrients (Lindenmayer et al. 2012; Lindenmayer and Laurance 2016). At least one to two centuries are needed for the recovery of large trees to enhance the forest ecosystem's diversity, architectural structure and functions (Bonnell et al. 2011; Lindenmayer et al. 2012; Chazdon 2014). In our study sites, planted trees gained a DBH of 20 cm at approximately 14 to 21 years after planting (Appendix S2; Figure S2). Also, Ssekuubwa et al. (2022) showed a small but significant increase in many structural and diversity variables for large trees (there, DBH ≥ 30 cm) in the same actively restored area as our study, between 2003 and 2017.

### 4.2 Enhanced Tree Diversity and Structure—A Sign of Seed Dispersal Recovery?

Tree diversity and forest structure increased gradually along the age gradient, implying that active restoration has prompted a crucial ecosystem function—seed dispersion. In our restoration project area in Kibale, some previously deforested areas have drifted to an arrested succession state (i.e., transformed into grasslands) where tree establishment is virtually absent compared to actively restored sites (Wheeler et al. 2016; UWA‐FACE 2015). In these grasslands, Wheeler et al. (2016) found very low tree species richness and no trees (DBH ≥ 10 cm). Stem density of seedlings was extremely low—1% seedling cover in grasslands (sampled 21 years after the end of disturbance) compared to 5% in an actively restored forest (10 years since planting). Their results propose that seed dispersion and/or seedling survival were almost non‐existent in deforested areas unable to regenerate naturally. Hence, we conclude that tree planting has enhanced forest recovery and seed dispersion in our study sites, which had the same initial conditions as those grasslands in Wheeler et al. (2016) but were eventually planted. Although around 60 species have established in the restored forests (Appendix S1: Table S1), this is only a fraction of the > 350 tree species recorded in Kibale's primary forest (Howard et al. 1996). Thus, numerous later‐successional tree species could still potentially colonise the restoration area.

Several factors can facilitate or filter seed dispersal and tree establishment during forest recovery at our restoration study sites. Seeds are dispersed via wind or complex groups of animals. Factors affecting seed dispersal include (1) the distance between the source population and the restoration area, (2) the attractiveness of the area to seed dispersers and (3) the diversity of seed dispersers (Lindell et al. 2013; Chazdon 2014; Reid et al. 2015). Firstly, in our study area, the distance between the primary forest and the restoration sites varied between 500 and 6000 m, which was shown to affect the community compositions. Therefore, the distance between some of the most remote restored forests and the primary forest edge might hinder the dispersal and recovery of the tree community (Zahawi et al. 2021; Camargo et al. 2020).

Secondly, structurally and taxonomically diverse tree communities attract forest‐associated seed dispersers to roost and feed (Chazdon 2014; Reid et al. 2015). However, relatively young, restored forests might not be alluring for all larger seed dispersers, as the forest structure is still developing (Lindell et al. 2013; Chazdon 2014) and resources or conditions that the dispersers require are inadequate. Nevertheless, the structure and the tree diversity of the studied restored forests have progressively shifted from grasslands to (young) successional forests. This indicates that at some point, restored forests have become more attractive to seed dispersers; the forest structure has changed, the tree taxa richness has increased and most of the species are animal‐dispersed.

Thirdly, the seed‐dispersing animal community affects whether certain trees can colonise the restored areas. For example, large and specialised animals usually distribute large‐fruited and/or seeded late‐successional trees (Chazdon 2014; Pohlman et al. 2021). In Kibale, important dispersers include elephants ( *Loxodonta africana* ), birds and primates. Elephants and some primate species have already colonised the restored compartments (Omeja et al. 2011; Ssekuubwa et al. 2018; van Goor 2021). Furthermore, the abundance of forest specialist birds tends to increase along the restoration age gradient (Latja et al. 2016). These dispersers can change the composition of seed rain arriving in different‐aged restored forests. Therefore, proximity to primary forests may enable a positive feedback loop: as the restored forests grow and get more complex, they can lure a more diverse community of seed dispersers, who might bring seeds of new (late‐successional) tree species and enrich the forest community, structure and functions.

Duncan and Chapman (1999) found that 2–3 years post‐disturbance, less than 1% of the tree seeds were forest‐dependent species in the degraded forests that were turned into grasslands in Kibale. In contrast, Jacob et al. (2017) discovered that 18–21 years after the disturbance had ended in Kibale, a third of the stems growing under or near remnant trees were forest‐dependent. Our results showed that at least four late‐successional (i.e., animal‐dispersed, forest‐interior and shade‐tolerant) taxa have dispersed to restored forests from the primary forest after 13–26 years of planting. These findings support the conclusion that late‐successional trees spread very slowly to the restoration area, experiencing probably both dispersal and establishment limitations.

### 4.3 Slow Turnover in Community Composition

After 20 years of planting, the composition of the restored tree communities is still distinct from that of the primary forest, as we expected. This indicates that community recovery is likely to be a slow process, as suggested by many earlier studies in similar systems. For example, Poorter et al. (2021) estimate that the community composition needs at least 120 years to recover 90% of reference site values in Neo‐ and Afrotropical forests. Rozendaal et al. (2019) concluded that the recovery of the community composition of secondary Neotropical forests may span from decades to several centuries.

Inspecting the community composition according to the tree size revealed that sapling and small tree communities of the restored forests are moving towards the primary forest compositions. Our results also suggest species turnover can be relatively swift for saplings and small trees. No such changes were observed among the large‐sized tree communities, probably because their abundance is low, and the time scale (8 years) is relatively short. Previously, Ssekuubwa et al. (2022) observed similar patterns in Kibale; the compositional similarity of small‐sized and medium‐sized trees in restored forests vs. primary forest was higher than that of large trees. Even though the small trees should not be used solely for monitoring the forest recovery process (Ssekuubwa et al. 2022), including them gives additional information about the recovery process. After all, the seedling and sapling community creates the potential for the future tree community. Moreover, they give complementary information, as the medium and large tree classes still mostly represent what has been planted (when the survey is done two to three decades after planting).

### 4.4 Changes Occurring in the Primary Forest

In the primary forest reference sites, the observed tree taxa richness and basal area were lower in 2021 than in the 2013 survey. As explained above, we were unable to locate the plots exactly in the same position in 2021 as in 2013 because the plot orientation was not recorded in 2013. Therefore, the set of sampled tree stems differed over the survey years, and the exclusion of some of the larger trees (based on DBH) measured in 2013 might partly explain the lower total basal area in 2021. Another possible explanation could be stochastic processes in the natural tree mortality occurring over the 8 years. Increased tree mortality has been reported to occur in Kibale at the end of the 20th century (Lwanga 2003), but we found no new mass mortality reports in recent years.

### 4.5 Is Tree Planting Facilitating Forest Recovery?

Even though we had no control study sites from the nearby unplanted grasslands, studies from our research area have previously shown that without active restoration, tree establishment is unsuccessful (Wheeler et al. 2016; Duncan and Duncan 2000). Our results showed gradual but partial recovery in many forest structure and diversity characteristics and a large variation in the variables among some study sites within the same planting year (Figure 2; Appendix S1: Figure S3). Partial recovery is expected because this work describes the early phases of rainforest succession after active restoration; full recovery of the rainforest structure, diversity and functions would take at least a century (Liebsch et al. 2008; Bonnell et al. 2011; Chazdon 2014; Poorter et al. 2021). The first years of secondary succession (i.e., the stand initiation phase) are crucial in determining how succession starts. In the stand initiation phase, multiple successional paths are “open” and dependent on the restorative practices, the land‐use history and other biotic and abiotic factors specific to the site and surrounding landscape (Arroyo‐Rodríguez et al. 2017; Jakovac et al. 2021).

Based on previous studies, the key to successful active forest restoration is the use of “framework species”, i.e., species that are native, can tolerate both competing vegetation and exposed conditions, and can transform the environment to be more attractive to seed dispersers (reviewed by Elliott et al. 2022). In our study, *B. micrantha* and *S. elliptica* stand out as framework species, fulfilling the criteria stated above (Appendix S2: Figure S2; see also Wheeler et al. 2016). The restoration planting has been generally effective with these species; only a few of our study sites (planted in 2005, 2008 and 2011; Appendix S1: Figure S3) had no, or only a couple of, planted tree stems surviving during our surveys. Reasons for the planting failures in our study area in these few cases could be related to stochastic drivers such as diseases, herbivore damage, or unfavourable soil conditions (Höhl et al. 2020). Although arrested succession can occur in actively restored areas, our results indicate that planting lowers the probability of it.

The compositional changes in the restored forest were similar whether planted trees were included or excluded from the analyses (Appendix S1: Tables S3 and S4). This indicates that the age gradient in the community compositions is significant among the non‐planted trees (i.e., the naturally regenerating element of the tree compositions) across the restoration area and is not produced by the temporal variation in the composition of planted trees. In summary, although only seven planted species established themselves on our study plots, the planting enabled forest recovery at the very start of the restoration process.

## Author Contributions

**Eveliina Korkiatupa:** conceptualization (equal), data curation (equal), formal analysis (lead), methodology (supporting), visualization (lead), writing – original draft (lead), writing – review and editing (equal). **Geoffrey M. Malinga:** resources (equal), writing – original draft (supporting), writing – review and editing (equal). **Sille Holm:** conceptualization (equal), investigation (equal), supervision (equal), writing – original draft (supporting), writing – review and editing (equal). **Wouter van Goor:** funding acquisition (supporting), resources (equal), writing – review and editing (equal). **Richard Kigenyi:** funding acquisition (supporting), resources (equal), writing – review and editing (equal). **Anu Valtonen:** conceptualization (equal), data curation (equal), formal analysis (supporting), funding acquisition (lead), investigation (equal), methodology (lead), project administration (lead), supervision (equal), visualization (supporting), writing – original draft (supporting), writing – review and editing (equal).

## Conflicts of Interest

W.V.G. works at Face the Future as a forest project manager. R.K. works at Uganda Wildlife Authority as Warden of Forest Restoration. The remaining authors declare no conflicts of interest.

## Supporting information


Appendix S1.

Appendix S2.

## Data Availability

The data (Korkiatupa et al. 2025) supporting this study's findings is available in the Dryad repository: https://doi.org/10.5061/dryad.z34tmpgsk.

## References

[ece372033-bib-0001] Abiem, I. , I. Dickie , D. Kenfack , and H. M. Chapman . 2023. “Factors Limiting Plant Recruitment in a Tropical Afromontane Forest.” Biotropica 55, no. 1: 221–231. 10.1111/btp.13179.

[ece372033-bib-0002] Aleman, J. C. , M. A. Jarzyna , and A. C. Staver . 2017. “Forest Extent and Deforestation in Tropical Africa Since 1900.” Nature Ecology & Evolution 2, no. 1: 26–33. 10.1038/s41559-017-0406-1. 29230024

[ece372033-bib-0003] Anderson, M. J. , R. N. Gorley , and K. R. Clarke . 2008. PERMANOVA+ for PRIMER: Guide to Sofware and Statistical Methods. Primer‐E Ltd.

[ece372033-bib-0004] Arroyo‐Rodríguez, V. , F. P. L. Melo , M. Martínez‐Ramos , et al. 2017. “Multiple Successional Pathways in Human‐Modified Tropical Landscapes: New Insights From Forest Succession, Forest Fragmentation and Landscape Ecology Research: Multiple Successional Pathways.” Biological Reviews 92, no. 1: 326–340. 10.1111/brv.12231. 26537849

[ece372033-bib-0005] Bonnell, T. R. , R. Reyna‐Hurtado , and C. A. Chapman . 2011. “Post‐Logging Recovery Time Is Longer Than Expected in an East African Tropical Forest.” Forest Ecology and Management 261, no. 4: 855–864. 10.1016/j.foreco.2010.12.016.

[ece372033-bib-0006] Boulton, C. A. , T. M. Lenton , and N. Boers . 2022. “Pronounced Loss of Amazon Rainforest Resilience Since the Early 2000s.” Nature Climate Change 12, no. 3: 271–278. 10.1038/s41558-022-01287-8.

[ece372033-bib-0007] Camargo, P. H. S. A. , M. A. Pizo , P. H. S. Brancalion , and T. A. Carlo . 2020. “Fruit Traits of Pioneer Trees Structure Seed Dispersal Across Distances on Tropical Deforested Landscapes: Implications for Restoration.” Journal of Applied Ecology 57, no. 12: 2329–2339. 10.1111/1365-2664.13697.

[ece372033-bib-0008] Chapman, C. A. , C. Galán‐Acedo , J. F. Gogarten , et al. 2021. “A 40‐Year Evaluation of Drivers of African Rainforest Change.” Forest Ecosystems 8, no. 1: 66. 10.1186/s40663-021-00343-7.

[ece372033-bib-0009] Chapman, C. A. , and J. E. Lambert . 2000. “Habitat Alteration and the Conservation of African Primates: Case Study of Kibale National Park, Uganda.” American Journal of Primatology 50, no. 3: 169–185. 10.1002/(SICI)1098-2345(200003)50:3<169::AID-AJP1>3.0.CO;2-P. 10711532

[ece372033-bib-0010] Chazdon, R. L. 2008. “Beyond Deforestation: Restoring Forests and Ecosystem Services on Degraded Lands.” Science 320, no. 5882: 1458–1460. 10.1126/science.1155365. 18556551

[ece372033-bib-0011] Chazdon, R. L. 2014. Second Growth: The Promise of Tropical Forest Regeneration in an Age of Deforestation, 449. University of Chicago Press. 10.7208/chicago/9780226118109.001.0001.

[ece372033-bib-0012] Clarke, K. R. , and R. N. Gorley . 2006. Primer v6: User Manual/Tutorial. Primer‐E Ltd.

[ece372033-bib-0014] Crouzeilles, R. , M. S. Ferreira , R. L. Chazdon , et al. 2017. “Ecological Restoration Success Is Higher for Natural Regeneration Than for Active Restoration in Tropical Forests.” Science Advances 3, no. 11: e1701345. 10.1126/sciadv.1701345. 29134195 PMC5677348

[ece372033-bib-0015] Di Sacco, A. , K. A. Hardwick , D. Blakesley , et al. 2021. “Ten Golden Rules for Reforestation to Optimize Carbon Sequestration, Biodiversity Recovery and Livelihood Benefits.” Global Change Biology 27, no. 7: 1328–1348. 10.1111/gcb.15498. 33494123

[ece372033-bib-0016] Duclos, V. , S. Boudreau , and C. A. Chapman . 2013. “Shrub Cover Influence on Seedling Growth and Survival Following Logging of a Tropical Forest.” Biotropica 45, no. 4: 419–426. 10.1111/btp.12039.

[ece372033-bib-0017] Duncan, R. S. , and C. A. Chapman . 1999. “Seed Dispersal and Potential Forest Succession in Abandoned Agriculture in Tropical Africa.” Ecological Applications 9, no. 3: 998–1008. 10.1890/1051-0761(1999)009[0998:SDAPFS]2.0.CO;2.

[ece372033-bib-0018] Duncan, R. S. , and V. E. Duncan . 2000. “Forest Succession and Distance From Forest Edge in an Afro‐Tropical Grassland^1^ .” Biotropica 32, no. 1: 33–41. 10.1111/j.1744-7429.2000.tb00445.x.

[ece372033-bib-0019] Elliott, S. , D. Blakesley , and K. Hardwick . 2013. Restoring Tropical Forests: A Practical Guide, 344. Royal Botanic Gardens.

[ece372033-bib-0020] Elliott, S. , N. I. J. Tucker , D. P. Shannon , and P. Tiansawat . 2022. “The Framework Species Method: Harnessing Natural Regeneration to Restore Tropical Forest Ecosystems.” Philosophical Transactions of the Royal Society, B: Biological Sciences 378, no. 1867: 20210073. 10.1098/rstb.2021.0073. PMC9661958 36373920

[ece372033-bib-0021] Estrada‐Villegas, S. , P. R. Stevenson , O. López , S. J. DeWalt , L. S. Comita , and D. H. Dent . 2022. “Animal Seed Dispersal Recovery During Passive Restoration in a Forested Landscape.” Philosophical Transactions of the Royal Society, B: Biological Sciences 378: 20210076. 10.1098/rstb.2021.0076. PMC9661942 36373921

[ece372033-bib-0022] Ferreira, M. C. , and D. L. M. Vieira . 2024. “Making the Most of Native Seeds: Management Techniques Interact With Seed and Seedling Traits for Enhancing Direct Seeding Success.” Forest Ecology and Management 574: 122353. 10.1016/j.foreco.2024.122353.

[ece372033-bib-0023] Flores, B. M. , and M. Holmgren . 2021. “Why Forest Fails to Recover After Repeated Wildfires in Amazonian Floodplains? Experimental Evidence on Tree Recruitment Limitation.” Journal of Ecology 109, no. 10: 3473–3486. 10.1111/1365-2745.13769.

[ece372033-bib-0024] Gann, G. D. , T. McDonald , B. Walder , et al. 2019. “International Principles and Standards for the Practice of Ecological Restoration. Second Edition.” Restoration Ecology 27, no. S1: S1–S46. 10.1111/rec.13035.

[ece372033-bib-0025] Ghazoul, J. , and D. Sheil . 2010. Tropical Rain Forest Ecology, Diversity, and Conservation, 516. Oxford University Press.

[ece372033-bib-0026] Guariguata, M. R. , and R. Ostertag . 2001. “Neotropical Secondary Forest Succession: Changes in Structural and Functional Characteristics.” Forest Ecology and Management 148, no. 1–3: 185–206. 10.1016/S0378-1127(00)00535-1.

[ece372033-bib-0027] Hartter, J. , S. J. Ryan , J. Southworth , and C. A. Chapman . 2011. “Landscapes as Continuous Entities: Forest Disturbance and Recovery in the Albertine Rift Landscape.” Landscape Ecology 26, no. 6: 877–890. 10.1007/s10980-011-9616-0.

[ece372033-bib-0028] Hawthorne, W. D. 1995. Ecological Profiles of Ghanaian Forest Trees, 345. Oxford Forestry Institute, Department of Plant Sciences, University of Oxford [u.a.] (Tropical forestry papers, 29).

[ece372033-bib-0029] Höhl, M. , V. Ahimbisibwe , J. A. Stanturf , P. Elsasser , M. Kleine , and A. Bolte . 2020. “Forest Landscape Restoration—What Generates Failure and Success?” Forests 11, no. 9: 938. 10.3390/f11090938.

[ece372033-bib-0030] Holl, K. D. , J. L. Reid , J. M. Chaves‐Fallas , F. Oviedo‐Brenes , and R. A. Zahawi . 2017. “Local Tropical Forest Restoration Strategies Affect Tree Recruitment More Strongly Than Does Landscape Forest Cover.” Journal of Applied Ecology 54, no. 4: 1091–1099. 10.1111/1365-2664.12814.

[ece372033-bib-0031] Hooper, E. , R. Condit , and P. Legendre . 2002. “Responses of 20 Native Tree Species to Reforestation Strategies for Abandoned Farmland in Panama.” Ecological Applications 12, no. 6: 1626–1641. 10.1890/1051-0761(2002)012[1626:RONTST]2.0.CO;2.

[ece372033-bib-0032] Howard, P. , T. Davenport , and R. Matthews . 1996. Kibale National Park Biodiversity Report. Uganda Forest Department.

[ece372033-bib-0033] Hsieh, T. C. , K. H. Ma , and A. Chao . 2016. “iNEXT: An R Package for Rarefaction and Extrapolation of Species Diversity (Hill Numbers).” Methods in Ecology and Evolution 7: 1451–1456. 10.1111/2041-210X.12613.

[ece372033-bib-0013] IBM Corp . 2023. IBM SPSS Statistics for Windows, Version 29.0.2.0. IBM Corp.

[ece372033-bib-0034] IPBES . 2019. Global Assessment Report of the Intergovernmental Science‐Policy Platform on Biodiversity and Ecosystem Services, edited by E. S. Brondízio , J. Settele , S. Díaz , and H. T. Ngo , 1144. IPBES Secretariat.

[ece372033-bib-0035] Jacob, A. L. , M. J. Lechowicz , and C. A. Chapman . 2017. “Non‐Native Fruit Trees Facilitate Colonization of Native Forest on Abandoned Farmland.” Restoration Ecology 25, no. 2: 211–219. 10.1111/rec.12414.

[ece372033-bib-0036] Jakovac, C. C. , A. B. Junqueira , R. Crouzeilles , M. Peña‐Claros , R. C. G. Mesquita , and F. Bongers . 2021. “The Role of Land‐Use History in Driving Successional Pathways and Its Implications for the Restoration of Tropical Forests.” Biological Reviews 96, no. 4: 1114–1134. 10.1111/brv.12694. 33709566 PMC8360101

[ece372033-bib-0037] Joyce, F. H. , B. M. Ramos , R. A. Zahawi , and K. D. Holl . 2024. “Vertebrate Seed Predation Can Limit Recruitment of Later‐Successional Species in Tropical Forest Restoration.” Biotropica 56, no. 6: e13381. 10.1111/btp.13381.

[ece372033-bib-0038] Kalema, J. , and A. Hamilton . 2020. Field Guide to the Forest Trees of Uganda: For Identification and Conservation, 277. CABI.

[ece372033-bib-0039] Korkiatupa, E. , G. M. Malinga , S. Holm , W. van Goor , R. Kigenyi , and A. Valtonen . 2025. “Data From: Chronosequence Resampling Elucidates Tree Community and Forest Structure Recovery Patterns in Restored Tropical Rainforest, Dryad.” 10.5061/dryad.z34tmpgsk. PMC12375826 40860228

[ece372033-bib-0040] Kulikowski, A. J. , R. A. Zahawi , L. K. Werden , K. Zhu , and K. D. Holl . 2022. “Restoration Interventions Mediate Tropical Tree Recruitment Dynamics Over Time.” Philosophical Transactions of the Royal Society, B: Biological Sciences 378, no. 1867: 20210077. 10.1098/rstb.2021.0077. PMC9661957 36373915

[ece372033-bib-0041] Lamb, D. , P. D. Erskine , and J. A. Parrotta . 2005. “Restoration of Degraded Tropical Forest Landscapes.” Science 310, no. 5754: 1628–1632. 10.1126/science.1111773. 16339437

[ece372033-bib-0042] Latja, P. , A. Valtonen , G. M. Malinga , and H. Roininen . 2016. “Active Restoration Facilitates Bird Community Recovery in an Afrotropical Rainforest.” Biological Conservation 200: 70–79. 10.1016/j.biocon.2016.05.035.

[ece372033-bib-0043] Lawes, M. J. , and C. A. Chapman . 2006. “Does the Herb Acanthus Pubescens and/or Elephants Suppress Tree Regeneration in Disturbed Afrotropical Forest?” Forest Ecology and Management 221, no. 1–3: 278–284. 10.1016/j.foreco.2005.10.039.

[ece372033-bib-0044] Liebsch, D. , M. C. M. Marques , and R. Goldenberg . 2008. “How Long Does the Atlantic Rain Forest Take to Recover After a Disturbance? Changes in Species Composition and Ecological Features During Secondary Succession.” Biological Conservation 141, no. 6: 1717–1725. 10.1016/j.biocon.2008.04.013.

[ece372033-bib-0045] Lindell, C. A. , J. L. Reid , and R. J. Cole . 2013. “Planting Design Effects on Avian Seed Dispersers in a Tropical Forest Restoration Experiment.” Restoration Ecology 21, no. 4: 515–522. 10.1111/j.1526-100X.2012.00905.x.

[ece372033-bib-0046] Lindenmayer, D. B. , and W. F. Laurance . 2016. “The Unique Challenges of Conserving Large Old Trees.” Trends in Ecology & Evolution 31, no. 6: 416–418. 10.1016/j.tree.2016.03.003. 27117523

[ece372033-bib-0047] Lindenmayer, D. B. , W. F. Laurance , and J. F. Franklin . 2012. “Global Decline in Large Old Trees.” Science 338: 1305. https://foi.org/10.1126/science‐1231070. 23224548 10.1126/science.1231070

[ece372033-bib-0048] Lutz, J. A. , T. J. Furniss , D. J. Johnson , et al. 2018. “Global Importance of Large‐Diameter Trees.” Global Ecology and Biogeography 27, no. 7: 849–864. 10.1111/geb.12747.

[ece372033-bib-0049] Lwanga, J. S. 2003. “Localized Tree Mortality Following the Drought of 1999 at Ngogo, Kibale National Park, Uganda.” African Journal of Ecology 41, no. 2: 194–196. 10.1046/j.1365-2028.2003.00428.x.

[ece372033-bib-0050] Magurran, A. E. , and B. J. McGill . 2011. Biological Diversity: Frontiers in Measurement and Assessment, 345. Oxford University Press.

[ece372033-bib-0051] Mantoani, M. C. , and J. M. D. Torezan . 2016. “Regeneration Response of Brazilian Atlantic Forest Woody Species to Four Years of *Megathyrsus maximus* Removal.” Forest Ecology and Management 359: 141–146. 10.1016/j.foreco.2015.10.004.

[ece372033-bib-0052] Maurent, E. , B. Hérault , C. Piponiot , et al. 2023. “A Common Framework to Model Recovery in Disturbed Tropical Forests.” Ecological Modelling 483: 110418. 10.1016/j.ecolmodel.2023.110418.

[ece372033-bib-0053] Meli, P. , K. D. Holl , J. M. Rey Benayas , et al. 2017. “A Global Review of Past Land Use, Climate, and Active vs. Passive Restoration Effects on Forest Recovery.” PLoS One 12, no. 2: e0171368. 10.1371/journal.pone.0171368. 28158256 PMC5291368

[ece372033-bib-0054] Mesquita, R. D. C. G. , P. E. D. S. Massoca , C. C. Jakovac , T. V. Bentos , and G. B. Williamson . 2015. “Amazon Rain Forest Succession: Stochasticity or Land‐Use Legacy?” Bioscience 65, no. 9: 849–861. 10.1093/biosci/biv108.

[ece372033-bib-0055] Natural Earth . 2023. Downloads. Natural Earth Accessed August 1, 2023. www.naturalearthdata.com/downloads/.

[ece372033-bib-0056] Nyafwono, M. , A. Valtonen , P. Nyeko , and H. Roininen . 2014. “Butterfly Community Composition Across a Successional Gradient in a Human‐Disturbed Afro‐Tropical Rain Forest.” Biotropica 46, no. 2: 210–218. 10.1111/btp.12085.

[ece372033-bib-0057] Omeja, P. A. , C. A. Chapman , J. Obua , et al. 2011. “Intensive Tree Planting Facilitates Tropical Forest Biodiversity and Biomass Accumulation in Kibale National Park, Uganda.” Forest Ecology and Management 261, no. 3: 703–709. 10.1016/j.foreco.2010.11.029.

[ece372033-bib-0058] Omeja, P. A. , M. J. Lawes , A. Corriveau , et al. 2016. “Recovery of Tree and Mammal Communities During Large‐Scale Forest Regeneration in Kibale National Park, Uganda.” Biotropica 48, no. 6: 770–779. 10.1111/btp.12360.

[ece372033-bib-0059] Osuri, A. M. , D. Mudappa , S. Kasinathan , and T. R. S. Raman . 2022. “Canopy Cover and Ecological Restoration Increase Natural Regeneration of Rainforest Trees in the Western Ghats, India.” Restoration Ecology 30, no. 5: e13558. 10.1111/rec.13558.

[ece372033-bib-0060] Patel, N. 2018. “CanopyCapture.” Accessed January 23, 2023. https://nikp29.github.io/CanopyCapture/.

[ece372033-bib-0061] Peña‐Domene, M. D. L. , C. Martínez‐Garza , L. M. Ayestarán‐Hernández , and H. F. Howe . 2018. “Plant Attributes That Drive Dispersal and Establishment Limitation in Tropical Agricultural Landscapes.” Forests 9, no. 10: 620. 10.3390/f9100620.

[ece372033-bib-0062] Piiroinen, T. , A. Valtonen , and H. Roininen . 2017. “The Seed‐To‐Seedling Transition Is Limited by Ground Vegetation and Vertebrate Herbivores in a Selectively Logged Rainforest.” Forest Ecology and Management 384: 137–146. 10.1016/j.foreco.2016.10.037.

[ece372033-bib-0063] Pohlman, C. L. , D. Y. P. Tng , and S. K. Florentine . 2021. “Do Primary Rainforest Tree Species Recruit Into Passively and Actively Restored Tropical Rainforest?” Forest Ecology and Management 496: 119453. 10.1016/j.foreco.2021.119453.

[ece372033-bib-0064] Poorter, L. , D. Craven , C. C. Jakovac , et al. 2021. “Multidimensional Tropical Forest Recovery.” Science 374, no. 6573: 1370–1376. 10.1126/science.abh3629. 34882461

[ece372033-bib-0065] QGIS . 2021. QGIS Geographic Information System. QGIS Association. http://www.qgis.org.

[ece372033-bib-0066] R Core Team . 2023. R: A Language and Environment for Statistical Computing. R Foundation for Statistical Computing. https://www.r‐project.org/.

[ece372033-bib-0067] Regional Centre for Mapping of Resources for Development . 2018. “Uganda SRTM DEM 30 Meters.” Accessed October 19, 2023. https://www.africageoportal.com/datasets/rcmrd::uganda‐srtm‐dem‐30‐meters/about.

[ece372033-bib-0068] Reid, J. L. , M. E. Fagan , and R. A. Zahawi . 2018. “Positive Site Selection Bias in Meta‐Analyses Comparing Natural Regeneration to Active Forest Restoration.” Science Advances 4, no. 5: eaas9143. 10.1126/sciadv.aas9143. 29774239 PMC5955619

[ece372033-bib-0069] Reid, J. L. , K. D. Holl , and R. A. Zahawi . 2015. “Seed Dispersal Limitations Shift Over Time in Tropical Forest Restoration.” Ecological Applications 25, no. 4: 1072–1082. 10.1890/14-1399.1. 26465043

[ece372033-bib-0070] Riemer, K. 2020. Verified Carbon Standard Monitoring Report 2017–2020, 59. Face the Future, Uganda Wildlife Authority Accessed September 13, 2024. https://registry.verra.org/app/projectDetail/VCS/673.

[ece372033-bib-0071] Rochimi, D. , K. Waring , and A. Sánchez Meador . 2021. “Evaluating Early Post‐Fire Tropical Lower Montane Forest Recovery in Indonesia.” Journal of Tropical Forest Science 33, no. 2: 113–125. 10.26525/jtfs2021.33.2.113.

[ece372033-bib-0072] Rodrigues, S. B. , M. G. Freitas , E. M. Campos‐Filho , et al. 2019. “Direct Seeded and Colonizing Species Guarantee Successful Early Restoration of South Amazon Forests.” Forest Ecology and Management 451: 117559. 10.1016/j.foreco.2019.117559.

[ece372033-bib-0073] Rozendaal, D. M. A. , F. Bongers , T. M. Aide , et al. 2019. “Biodiversity Recovery of Neotropical Secondary Forests.” Science Advances 5, no. 3: eaau3114. 10.1126/sciadv.aau3114. 30854424 PMC6402850

[ece372033-bib-0074] Sady, G. C. , K. D. Holl , R. J. Cole , and R. A. Zahawi . 2010. “Germination and Survival of Tree Seeds in a Tropical Montane Forest Restoration Study (Costa Rica).” Ecological Restoration 28, no. 2: 121–124. 10.3368/er.28.2.121.

[ece372033-bib-0075] Sangsupan, H. A. , D. E. Hibbs , B. A. Withrow‐Robinson , and S. Elliott . 2018. “Seed and Microsite Limitations of Large‐Seeded, Zoochorous Trees in Tropical Forest Restoration Plantations in Northern Thailand.” Forest Ecology and Management 419: 91–100. 10.1016/j.foreco.2018.03.021.

[ece372033-bib-0076] Shoo, L. P. , K. Freebody , J. Kanowski , and C. P. Catterall . 2016. “Slow Recovery of Tropical Old‐Field Rainforest Regrowth and the Value and Limitations of Active Restoration.” Conservation Biology 30, no. 1: 121–132. 10.1111/cobi.12606. 26310383

[ece372033-bib-0077] Skorupa, J. P. 1988. “The Effects of Selective Timber Harvesting on Rain‐Forest Primates in Kibale Forest, Uganda.” Doctoral dissertation. University of California. Accessed July 19, 2024. https://hdl.handle.net/2027/uc1.x32709.

[ece372033-bib-0078] Ssekuubwa, E. , L. E. Loe , D. Sheil , M. Tweheyo , and S. R. Moe . 2018. “Comparing Seed Removal Rates in Actively and Passively Restored Tropical Moist Forests.” Restoration Ecology 26, no. 4: 720–728. 10.1111/rec.12629.

[ece372033-bib-0079] Ssekuubwa, E. , W. Van Goor , M. Snoep , K. Riemer , F. Wanyama , and M. Tweheyo . 2021. “Recovery of Seedling Community Attributes During Passive Restoration of a Tropical Moist Forest in Uganda.” Applied Vegetation Science 24, no. 1: e12559. 10.1111/avsc.12559.

[ece372033-bib-0080] Ssekuubwa, E. , W. Van Goor , M. Snoep , et al. 2022. “Does Restoration Success Vary With Tree Size Under Restoration Plantings and Regrowth Forests?” Conservation Science and Practice 4, no. 9: e12781. 10.1111/csp2.12781.

[ece372033-bib-0081] Struhsaker, T. T. 1997. “Ecology of an African Rain Forest: Logging in Kibale and the Conflict Between Conservation and Exploitation.” Economic Botany 52: 80.

[ece372033-bib-0082] Suding, K. N. , and R. J. Hobbs . 2009. “Models of Ecosystem Dynamics as Frameworks for Restoration Ecology.” In New Models for Ecosystem Dynamics and Restoration, edited by R. J. Hobbs and K. N. Suding , 3–21. Island Press.

[ece372033-bib-0083] Swaine, M. D. , and T. C. Whitmore . 1988. “On the Definition of Ecological Species Groups in Tropical Rain Forests.” Vegetatio 75, no. 1–2: 81–86. 10.1007/BF00044629.

[ece372033-bib-0084] UWA‐FACE . 2015. Natural High Forest Rehabilitation Project on Degraded Land of Kibale National Park. CCB Project Design Document Accessed September 13, 2024. https://registry.verra.org/app/projectDetail/VCS/673.

[ece372033-bib-0085] van Breugel, M. , D. Craven , H. R. Lai , M. Baillon , B. L. Turner , and J. S. Hall . 2019. “Soil Nutrients and Dispersal Limitation Shape Compositional Variation in Secondary Tropical Forests Across Multiple Scales.” Journal of Ecology 107, no. 2: 566–581. 10.1111/1365-2745.13126.

[ece372033-bib-0086] van den Tweel, B. 2023. CCB MONITORING REPORT 2020–2023, 64. Face the Future, Uganda Wildlife Authority Accessed September 13, 2024. https://registry.verra.org/app/projectDetail/VCS/673.

[ece372033-bib-0087] van Goor, W. 2021. CCB Monitoring Report 2017–2020, 47. Face the Future, Uganda Wildlife Authority Accessed September 13, 2024. https://registry.verra.org/app/projectDetail/VCS/673.

[ece372033-bib-0088] Vancutsem, C. , F. Achard , J.‐F. Pekel , et al. 2021. “Long‐Term (1990–2019) Monitoring of Forest Cover Changes in the Humid Tropics.” Science Advances 7, no. 10: eabe1603. 10.1126/sciadv.abe1603. 33674308 PMC7935368

[ece372033-bib-0089] Wainwright, C. E. , T. L. Staples , L. S. Charles , et al. 2018. “Links Between Community Ecology Theory and Ecological Restoration Are on the Rise.” Journal of Applied Ecology 55, no. 2: 570–581. 10.1111/1365-2664.12975.

[ece372033-bib-0090] Werden, L. K. , K. D. Holl , J. A. Rosales , J. M. Sylvester , and R. A. Zahawi . 2020. “Effects of Dispersal‐ and Niche‐Based Factors on Tree Recruitment in Tropical Wet Forest Restoration.” Ecological Applications 30, no. 7: e02139. 10.1002/eap.2139. 32335980

[ece372033-bib-0091] Wheeler, C. E. , P. A. Omeja , C. A. Chapman , M. Glipin , C. Tumwesigye , and S. L. Lewis . 2016. “Carbon Sequestration and Biodiversity Following 18 Years of Active Tropical Forest Restoration.” Forest Ecology and Management 373: 44–55. 10.1016/j.foreco.2016.04.025.

[ece372033-bib-0092] Wing, L. D. , and I. O. Buss . 1970. “Elephants and Forests.” Wildlife Monographs 19: 3–92.

[ece372033-bib-0093] World Bank . 2018. Data Catalog—Africa Water Bodies. World Bank Accessed August 1, 2023. https://datacatalog.worldbank.org/search/dataset/0040797/Africa—Water‐Bodies.

[ece372033-bib-0094] Zahawi, R. A. , L. K. Werden , M. San‐José , J. A. Rosales , J. Flores , and K. D. Holl . 2021. “Proximity and Abundance of Mother Trees Affects Recruitment Patterns in a Long‐Term Tropical Forest Restoration Study.” Ecography 44, no. 12: 1826–1837. 10.1111/ecog.05907.

