# Supplementary material for: Chronosequence Resampling Elucidates Tree Community and Forest Structure Recovery Patterns in Restored Tropical Rainforest
Source: Ecol Evol. 2025 Aug 24;15(8):e72033. doi: 10.1002/ece3.72033 (PMC12375826; doi:10.1002/ece3.72033)
Supplement: Supplementary file 1 — Appendix S1. [file ECE3-15-e72033-s002.docx]

Ecology & Evolution

Appendix S1

**Title:** Chronosequence resampling elucidates tree community and forest structure recovery patterns in restored tropical rainforest

**Authors:** Eveliina Korkiatupa, Geoffrey M. Malinga, Sille Holm, Wouter van Goor, Richard Kigenyi, Anu Valtonen

**Vegetation surveys**


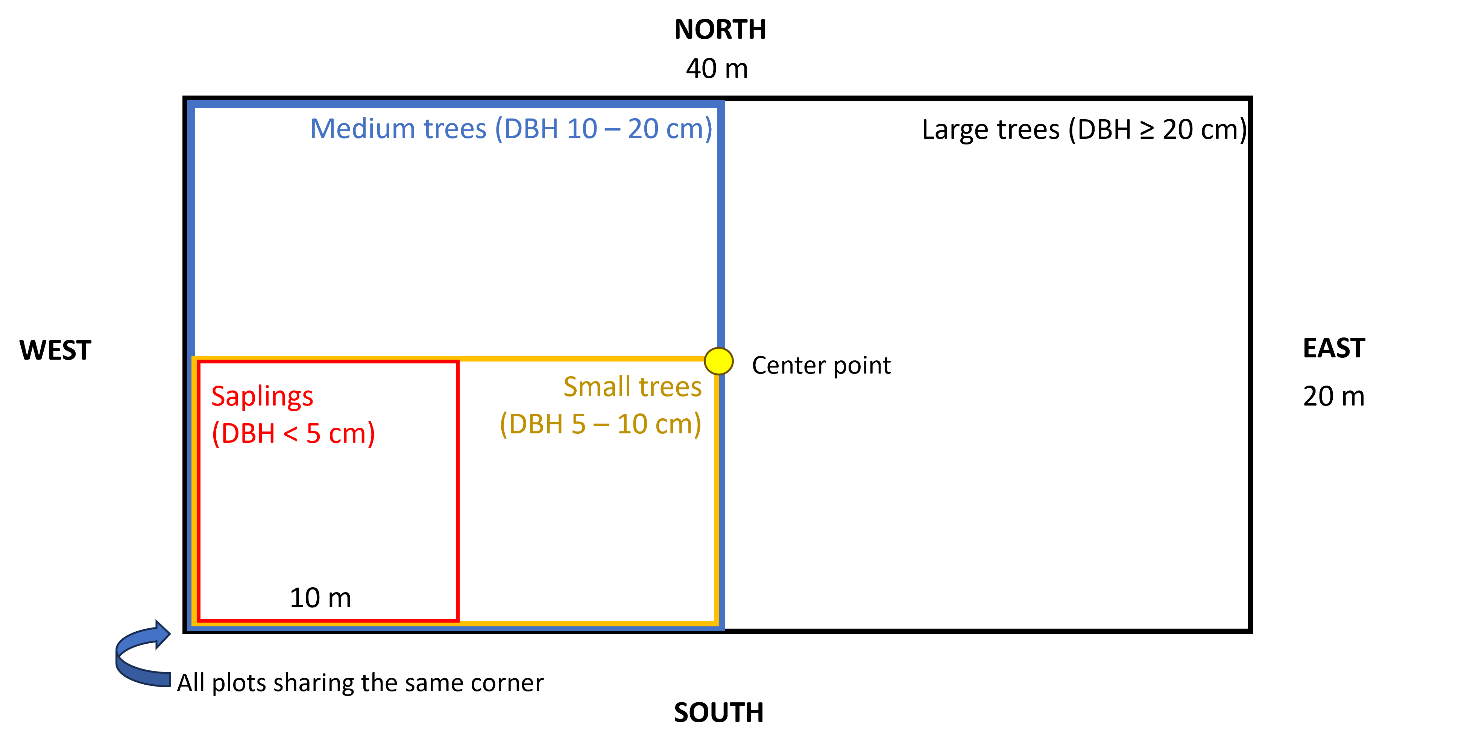


Figure S1. Nested study plot design of the vegetation survey in Kibale National Park. In the 2013 survey, only the coordinates of the corner (shared by sub-plots) were recorded (the orientation of the plot from the corner was not recorded). In the 2021 survey, we placed the centre of the plot in the known coordinates (the corner coordinates of the 2013 survey). This nested system is modified after Tabuti (2007) and used in our previous works (Nyafwono et al., 2015; Owiny et al., 2016).

*Unidentified taxa*

Altogether, 83% of the taxa represent species, 12% genera, and 5% remained unidentified. Unidentified tree species were listed as “Unknown 1”, “Unknown 2”, etc., for each study site, and they represented between 0% (in 2013) and 6% (in 2021) of all taxa. Unidentified species were included in all other analyses except the community multivariate analyses.

*Forest structure measurements*

The total estimated basal area was calculated based on the diameter at breast height (DBH) measurements. In each plot, the DBH (cm) of each large, medium and small tree was measured 1.3 m from the highest point at the ground at the tree’s base. If the tree was forked below 1.3 m, each branch’s DBH was measured and marked in the field notes. The tree canopy cover was measured from each of the four corners and the centre point of the plot with a phone app, *CanopyCapture* (Patel 2018). We will use the term “canopy cover” for simplicity reasons, even though the app measures the complement of canopy gap fraction (%), i.e., 100 - gap fraction (Lusk 2022).

Tree height (m) was measured with a phone app, *Trees* (Forest Monitoring Tools, 2023). To prepare for conditions, e.g. heavy rain, when using the phone app was not possible, field workers trained tree height measuring by eye with the phone app beforehand. The tree height was estimated by eye in 12 plots (26% of the plots in the restored forests). In each plot, the height was measured (estimated) from three planted trees closest to the centre point. The height was measured only from planted trees; although the restoration area has some large remnant trees, these were not included. In our final data, tree height information was missing from two study sites planted in 2011 and two study sites planted in 2017. Furthermore, we were unable to estimate the forest height for primary forest study sites, as seeing the treetops was difficult. Therefore, for the primary forest, we used a literature-based record of typical forest height (30 m) in Kibale (Wing & Buss 1970) as the reference point.

**Description of LMM models**

Linear mixed (effects) models (LMM) were fitted in SPSS (version 19.0.2.0 IBM Corp., 2023). Response variables were (1) tree taxa richness, (2) Simpson diversity, (3) total estimated basal area (m^2^/ha), (4) estimated stem density (stems/ha), (5) mean canopy cover (%), and (6) mean tree height (m). For each response variable we fitted a model where fixed (explanatory) variables were the survey time (two levels: 2013 & 2021), and the forest age group. The forest age group had three levels (intermediate-aged restored, older restored and primary forest). We also included the interaction term survey time × forest age group. Furthermore, we added study site (nested in planting year) as a random factor to the model. That is, the study sites were grouped by the planting year (pl) or belonging to the primary forest (pl1995, pl1999, pl2000, pl2005, pl2007, pl2008, primary forest). For the canopy cover (5) and tree height (6), the LMMs had only the forest age group as a fixed factor and no random term.

If the LMM detected differences in survey times or forest age groups, we executed pair-wise comparisons (LSD). Since many interaction terms (survey time × forest age group) were significant, the pair wise comparisons between forest age groups were done separately for each survey time.

**Additional information for data analyses**

*Tree traits*

We collected the information of traits from various sources (see the footnotes of Table S1). We have classified the dispersal type of some taxa either “animal”, “non-animal”, “animal assumed”, or “non-animal assumed”. If we were not able to find information of the dispersal type for certain species or genus, we assumed the dispersal type based on the literature of other species from the same genus. In case no information was found, or dispersal type varied within genus, we left the species undetermined (na). For Fisher’s exact tests (done in R; R Core Team, 2023), we grouped “animal” and “animal assumed” as one group and “non-animal” and “non-animal assumed” as other group. For all traits, we excluded the taxa where no information was available (i.e., na) from Fisher’s exact tests.

*DistLM*

For the 2013 DistLM model, omitting planted trees, we had to exclude five study sites with missing data on the status of planted trees (see above). For the 2021 DistLM model, one study site that had only planted trees (planted in 2008) had to be excluded from the analysis. We computed the distance to the primary forest as the shortest distance between the study site and the primary forest edge, following Valtonen et al. (2021), utilising the package “geosphere” (Hijmans et al., 2021) in R. The distance was rounded to 100 m.

**Results**

Table S1. List of tree taxa encountered in vegetation surveys in 2013 and 2021. Species names follow Plants of the World Online (<https://powo.science.kew.org/>).1 = present, 0 = absent. YR = younger restored (aged 4-10 years in 2021), IMR = intermediate-aged restored (aged 5–8 years in 2013, 13–16 years in 2021), OR = older restored (aged 13–18 years in 2013, 21–26 years in 2021), PF = primary forests. Habitat associations (as in Howard et al., 1996): F = forest interior, FE = forest edge, FG = forest generalist, R = riverine / lakeshore forest, WO = woodland, FN = forest-nondependent (occur in forest and open habitats). Seed dispersal types: animal = animal dispersed, non-ani = dispersion via wind, ballistic or gravity, animalA = animal dispersion assumed, non-aniA = dispersion via wind, ballistic, or gravity assumed. Seedling establishment guild (as in Hawthorne, 1995): pioneer = light demanders, npld = non-pioneer light demander, shade = shade tolerant, swamp = grows in swampy area, not ideal for other groups. na = when information was not available or assumptions could not be made.

|  |  | **2013** | | | **2021** | | | | **Habitat associations^1^** | **Seed dispersal type^2^** | **Seedling establishment guild^3^** |
| --- | --- | --- | --- | --- | --- | --- | --- | --- | --- | --- | --- |
| **Species** | **Author** | **IMR** | **OR** | **PF** | **YR** | **IMR** | **OR** | **PF** |  |  |  |
| *Albizia grandibracteata* | Taub. | 1 | 1 | 0 | 1 | 1 | 1 | 0 | FN | non-ani | npld |
| *Albizia gummifera*^abce^ | (J.F.Gmel.) C.A.Sm. | 0 | 1 | 0 | 0 | 1 | 1 | 0 | FG | non-ani | npld |
| *Albizia* sp1 |  | 0 | 0 | 0 | 1 | 0 | 0 | 1 | na | non-aniA | npld |
| *Alchornea laxiflora* | (Benth.) Pax & K.Hoffm. | 0 | 0 | 1 | 0 | 0 | 0 | 0 | FN | na | na |
| *Allophylus dummeri* | Baker f. | 0 | 1 | 0 | 0 | 0 | 0 | 0 | F | animal | shade |
| *Aningeria altissima*^ae^ | (A.Chev.) Aubrév. & Pellegr. | 1 | 0 | 1 | 0 | 0 | 1 | 0 | FG | animal | npld |
| *Antiaris toxicaria*^ae^ | (J.F.Gmel.) Lesch. | 0 | 0 | 1 | 0 | 1 | 0 | 1 | FN | animal | npld |
| *Balanites wilsoniana*^ae^ | Dawe & Sprague | 1 | 0 | 1 | 0 | 1 | 0 | 1 | F | animal | npld |
| *Bersama abyssinica* | Fresen. | 1 | 0 | 1 | 0 | 0 | 1 | 1 | FN | animal | pioneer |
| *Blighia unijugata*^e^ | Baker | 0 | 1 | 1 | 0 | 1 | 1 | 1 | FE | animal | shade |
| *Bridelia micrantha*^bcdf^ | (Hochst.) Baill. | 1 | 1 | 0 | 1 | 1 | 1 | 0 | FN | animal | pioneer |
| *Cassipourea ruwensorensis* | (Engl.) Alston | 0 | 1 | 1 | 0 | 0 | 1 | 1 | F | animal | shade |
| *Celtis africana* | Burm.f. | 0 | 1 | 1 | 0 | 0 | 1 | 1 | FG | animal | shade |
| *Celtis gomphophylla* | Baker | 1 | 1 | 1 | 0 | 1 | 1 | 1 | FG | animal | npld |
| *Chaetacme aristata* | Planch | 0 | 0 | 1 | 0 | 0 | 1 | 1 | FG | animal | shade |
| *Citropsis articulata* | (Willd. ex Spreng.) Swingle | 0 | 0 | 1 | 0 | 0 | 0 | 1 | F | animal | shade |
| *Clausena anisata* | (Willd.) Hook.f. ex Benth. | 0 | 1 | 1 | 0 | 0 | 1 | 1 | FN | animal | pioneer |
| *Coffea* sp. | L. | 0 | 1 | 1 | 0 | 0 | 0 | 1 | F | animal | shade |
| *Combretum molle* | R.Br. ex G.Don | 1 | 1 | 0 | 1 | 1 | 1 | 0 | WO | non-ani | npld |
| *Cordia africana*^ef^ | Lam. | 0 | 0 | 0 | 0 | 0 | 1 | 0 | FN | animal | pioneer |
| *Cordia millenii*^ef^ | Baker | 0 | 0 | 1 | 0 | 0 | 1 | 1 | F | animal | pioneer |
| *Crateva eminens* | (Hook.f.) Christenh. & Byng | 0 | 1 | 1 | 0 | 0 | 0 | 0 | F | non-ani | shade |
| *Croton* sp.^a-f^ | L. | 1 | 0 | 0 | 1 | 1 | 1 | 0 | na | animal | npld |
| *Cynometra alexandri* | C.H.Wright | 0 | 0 | 1 | 0 | 0 | 0 | 1 | na | non-ani | shade |
| *Dasylepis eggelingii* | J.B.Gillett | 0 | 0 | 1 | 0 | 0 | 0 | 1 | F | animal | shade |
| *Diospyros abyssinica* | (Hiern) F.White | 1 | 1 | 1 | 0 | 1 | 1 | 1 | FG | animal | shade |
| *Dombeya kirkii* | Mast. | 1 | 0 | 0 | 0 | 0 | 0 | 0 | FE | non-ani | pioneer |
| *Dovyalis macrocalyx* | (Oliv.) Warb. | 0 | 0 | 1 | 0 | 0 | 0 | 0 | FN | animal | npld |
| *Ehretia cymosa* | Thonn. | 0 | 0 | 1 | 0 | 0 | 0 | 0 | FN | animal | pioneer |
| *Englerophytum* sp. | K.Krause | 0 | 0 | 1 | 0 | 0 | 0 | 1 | F | animal | shade |
| *Entandrophragma angolense* | (Welw.) | 0 | 0 | 0 | 0 | 0 | 0 | 1 | F | non-ani | npld |
| *Erythrina abyssinica*^abce^ | Lam. | 1 | 1 | 0 | 0 | 1 | 1 | 0 | FN | non-ani | pioneer |
| *Fagaropsis angolensis*^ae^ | (Engl.) H.M.Gardner | 0 | 0 | 1 | 0 | 0 | 0 | 1 | FG | animal | npld |
| *Ficus asperifolia* | Miq. | 1 | 1 | 1 | 0 | 0 | 0 | 0 | FN | animal | shade |
| *Ficus saussureana* | DC. | 0 | 0 | 1 | 0 | 0 | 0 | 0 | FG | animal | swamp |
| *Ficus sur* | Forssk. | 1 | 0 | 0 | 0 | 0 | 0 | 0 | FN | animal | pioneer |
| *Ficus vallis-choudae* | Delile | 1 | 1 | 0 | 0 | 1 | 0 | 0 | R | animal | npld |
| *Funtumia* sp.^abce^ | Stapf | 1 | 1 | 1 | 0 | 1 | 1 | 1 | F | na | npld |
| *Gambeya* sp.^a-e^ | Pierre | 1 | 1 | 1 | 0 | 1 | 1 | 1 | F | animal | shade |
| *Harrisonia abyssinica* | Oliv. | 0 | 0 | 0 | 0 | 0 | 1 | 1 | F | animal | pioneer |
| *Harungana madagascariensis* | Lam. ex | 0 | 0 | 0 | 0 | 0 | 0 | 1 | F | animal | pioneer |
| *Hoslundia opposita* | Vahl. | 0 | 0 | 1 | 0 | 0 | 0 | 0 | na | animal | pioneer |
| *Kigelia africana* | (Lam.) Benth. | 0 | 1 | 1 | 1 | 1 | 1 | 0 | FN | animal | npld |
| *Lepisanthes senegalensis*^e^ | (Poir.) Leenh. | 0 | 1 | 1 | 0 | 1 | 0 | 1 | FN | animal | shade |
| *Leptactina arborescens*^a^ | (Welw. ex Benth. & Hook.f.) De Block | 0 | 0 | 1 | 0 | 0 | 0 | 1 | F | na | shade |
| *Leptonychia mildbraedii* | Engl. | 0 | 0 | 1 | 0 | 0 | 0 | 1 | F | na | shade |
| *Lovoa swynnertonii* | Baker f. | 0 | 0 | 1 | 0 | 0 | 1 | 1 | F | non-ani | npld |
| *Lychnodiscus cerospermus* | Radlk. | 0 | 0 | 1 | 0 | 0 | 0 | 1 | F | na | shade |
| *Macaranga* sp. | Thouars | 0 | 0 | 0 | 0 | 0 | 0 | 1 | na | animalA | pioneer |
| *Maesa lanceolata* | Forssk. | 1 | 1 | 0 | 0 | 1 | 0 | 0 | FN | animal | pioneer |
| *Maesopsis eminii* | Engl. | 0 | 1 | 0 | 0 | 1 | 1 | 0 | FN | animal | pioneer |
| *Mangifera indica* | L. | 0 | 0 | 0 | 1 | 0 | 0 | 0 | FN | animal | pioneer |
| *Margaritaria discoidea* | (Baill.) G.L.Webster | 0 | 0 | 1 | 0 | 0 | 1 | 1 | FN | animalA | pioneer |
| *Markhamia lutea*^a-e^ | (Benth.) K.Schum. | 0 | 1 | 1 | 0 | 0 | 1 | 0 | FE | non-ani | pioneer |
| *Maytenus undata* | (Thunb.) Blakelock | 0 | 0 | 1 | 0 | 0 | 0 | 0 | FE | animal | npld |
| *Millettia dura* | Dunn | 1 | 0 | 0 | 0 | 1 | 0 | 0 | FE | non-ani | npld |
| *Mimusops bagshawei*^a-e^ | S.Moore | 1 | 1 | 1 | 0 | 1 | 1 | 1 | F | animal | shade |
| *Monodora myristica*^e^ | (Gaertn.) Dunal | 1 | 1 | 1 | 0 | 1 | 1 | 1 | FG | animal | shade |
| *Myrianthus sp.* | P.Beauv. | 0 | 0 | 1 | 0 | 0 | 0 | 1 | na | animal | na |
| *Newtonia buchananii*^ae^ | (Baker) G.C.C.Gilbert & Boutique | 0 | 0 | 1 | 0 | 1 | 0 | 1 | FG | non-ani | npld |
| *Noronhia africana* | (Knobl.) Hong-Wa & Besnard | 0 | 0 | 1 | 0 | 1 | 1 | 0 | F | animal | shade |
| *Olea welwitschii* | (Knobl.) Gilg & G.Schellenb. | 0 | 1 | 0 | 0 | 0 | 1 | 0 | FG | animal | npld |
| *Oxyanthus speciosus* | DC. | 0 | 0 | 1 | 0 | 0 | 0 | 0 | F | animal | shade |
| *Pancovia turbinata*^a^ | Radlk. | 0 | 1 | 1 | 0 | 0 | 0 | 1 | F | animal | shade |
| *Parinari excelsa*^ae^ | Sabine | 0 | 0 | 1 | 0 | 0 | 0 | 0 | FG | animal | npld |
| *Persea americana* | Mill. | 1 | 0 | 0 | 0 | 0 | 0 | 0 | na | na | npld |
| *Pleiocarpa pycnantha* | (K.Schum.) Stapf | 0 | 0 | 1 | 0 | 0 | 0 | 0 | F | animal | swamp |
| *Premna angolensis* | Gürke | 0 | 0 | 1 | 0 | 0 | 1 | 0 | FE | animal | pioneer |
| *Prunus africana*^a-f^ | (Hook.f.) Kalkman | 1 | 1 | 0 | 0 | 1 | 1 | 0 | FN | animal | pioneer |
| *Pseudospondias microcarpa* | (A.Rich.) Engl. | 1 | 1 | 1 | 0 | 0 | 0 | 1 | FN | animal | swamp |
| *Psidium guajava* | L. | 0 | 1 | 0 | 1 | 0 | 1 | 0 | na | animal | pioneer |
| *Pterygota mildbraedii* | Engl. | 0 | 0 | 1 | 0 | 0 | 0 | 1 | FE | non-ani | npld |
| *Rauvolfia vomitoria* | Wennberg | 1 | 1 | 0 | 0 | 0 | 1 | 1 | FE | animal | pioneer |
| *Rinorea* sp. | Aubl. | 0 | 0 | 0 | 0 | 0 | 0 | 1 | F | na | shade |
| *Rothmannia* sp. | Thunb. | 0 | 1 | 1 | 0 | 0 | 1 | 1 | F | animal | shade |
| *Senna spectabilis* | (DC.) H.S.Irwin & Barneby | 0 | 0 | 0 | 1 | 1 | 1 | 0 | FN | na | pioneer |
| *Shirakiopsis elliptica*^abce^ | (Hochst.) Esser | 1 | 1 | 1 | 1 | 1 | 1 | 0 | FG | animal | pioneer |
| *Spathodea campanulata*^bdef^ | P.Beauv. | 1 | 1 | 0 | 0 | 1 | 1 | 0 | FE | non-ani | pioneer |
| *Strombosia scheffleri*^ae^ | Engl. | 0 | 0 | 1 | 0 | 0 | 0 | 1 | F | animal | shade |
| *Strychnos mitis* | S.Moore | 0 | 0 | 1 | 0 | 0 | 0 | 0 | FG | animal | shade |
| *Tabernaemontana* sp. | Plum. Ex L. | 1 | 1 | 1 | 0 | 1 | 1 | 1 | F | animal | shade |
| *Trema orientale* | (L.) Blume | 0 | 0 | 0 | 0 | 0 | 0 | 1 | FG | animal | pioneer |
| *Trichilia dregeana*^e^ | Sond. | 0 | 0 | 1 | 0 | 0 | 0 | 1 | F | animal | npld |
| *Trilepisium madagascarience* | DC. | 0 | 0 | 1 | 0 | 0 | 0 | 1 | FG | animal | npld |
| Unknown1 |  | 0 | 0 | 0 | 1 | 0 | 1 | 1 | na | na | na |
| Unknown2 |  | 0 | 0 | 0 | 0 | 0 | 1 | 1 | na | na | na |
| Unknown3 |  | 0 | 0 | 0 | 0 | 0 | 0 | 1 | na | na | na |
| Unknown4 |  | 0 | 0 | 0 | 0 | 0 | 0 | 1 | na | na | na |
| Unknown5 |  | 0 | 0 | 0 | 0 | 0 | 0 | 1 | na | na | na |
| *Uvariopsis congensis*^abcef^ | Robyns & Ghesq. | 1 | 1 | 1 | 0 | 1 | 1 | 1 | F | animal | shade |
| *Vachellia* sp. | Wight & Arn. | 1 | 1 | 0 | 1 | 1 | 1 | 0 | na | non-aniA | pioneer |
| *Vangueria apiculata* | K.Schum. | 0 | 0 | 1 | 0 | 0 | 0 | 1 | FE | animal | pioneer |
| *Vepris nobilis*^e^ | (Delile) Mziray | 0 | 0 | 1 | 0 | 0 | 1 | 1 | FN | animal | shade |
| *Warburgia ugandensis*^a-f^ | Sprague | 1 | 1 | 1 | 1 | 1 | 1 | 1 | FG | animal | npld |
| Total number of taxa |  | 30 | 38 | 59 | 13 | 31 | 44 | 54 |  |  |  |

^1^Sources: Howard et al. 1996; Kew Plants of the World (<https://powo.science.kew.org/>); Ssekuubwa et al. 2021; Kalema & Hamilton 2020.

^2^Sources: Hawthorne 1995; Lwanga 2003; Zanne et al. 2005; Loha et al. 2006; Babweteera 2009; Schleuning et al. 2011; Jacob et al. 2017; Ssekuubwa et al. 2021; Fujita et al. 2021.

^3^Sources: Hawthorne 1995; Sheil et al. 2000, 2006; Babweteera 2009; Jacob et al. 2017; Kalema & Hamilton 2020; Ssekuubwa et al. 2021.

^a-f^Used in planting years: a = 1995, b = 1997-1999, c = 2000, d = 2001-2009, e = 2011-2017, f = 2017-2020. Sources: unpublished FACE files; Wheeler et al. 2016; UWA-FACE 2015, 2017; van Goor 2021.


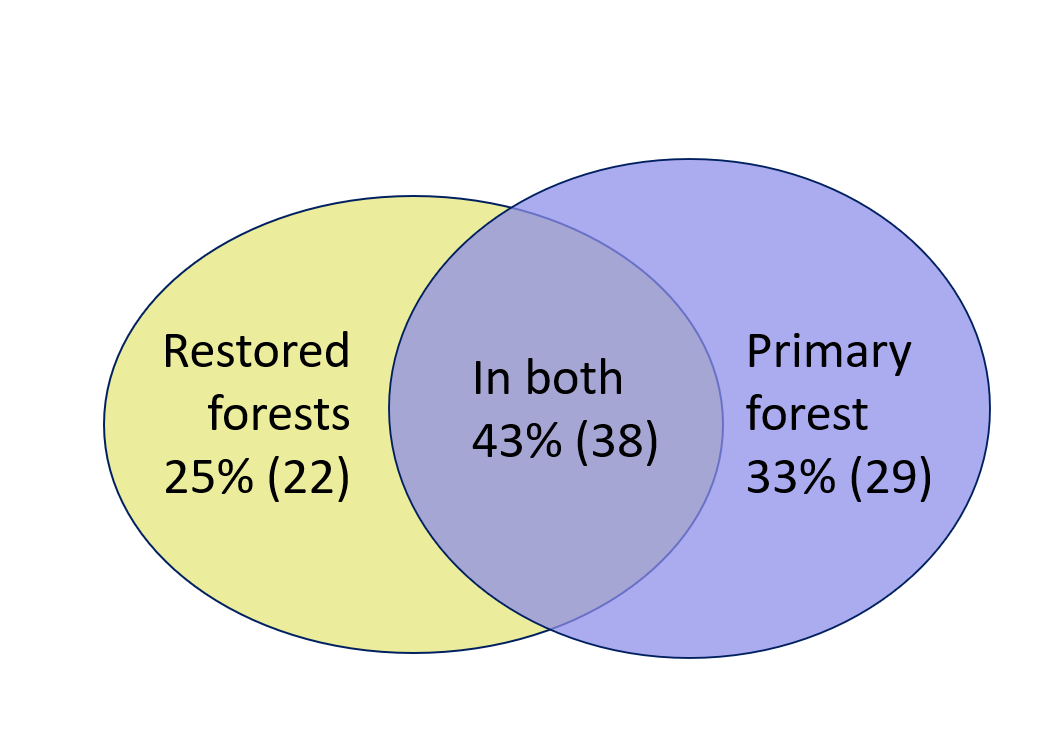


Figure S2. Venn diagram showing the percentages of the tree taxa encountered in restored and primary forests and both. Numbers in parenthesis show absolute numbers of identified taxa.

Table S2. The proportions of the five most common taxa based on the stem density (stems/ha).

| Survey year | Taxa | forest type | % of total stem density |
| --- | --- | --- | --- |
| 2013 | Bridelia micrantha | restored | 39.5 |
|  | Shirakiopsis elliptica | restored | 13.4 |
|  | Funtumia sp. | restored | 6.6 |
|  | Albizia grandibracteata | restored | 6.2 |
|  | Diospyros abyssinica | restored | 4.6 |
|  | Rest of the taxa | restored | 29.7 |
|  | Lovoa swynnertonii | primary | 27.6 |
|  | Uvariopsis congensis | primary | 9.0 |
|  | Pterygota mildbraedii | primary | 6.3 |
|  | Lepisanthes senegalensis | primary | 4.9 |
|  | Vepris nobilis | primary | 4.8 |
|  | Rest of the taxa | primary | 47.4 |
| 2021 | Funtumia sp. | restored | 21.4 |
|  | Bridelia micrantha | restored | 11.2 |
|  | Celtis gomphophylla | restored | 9.0 |
|  | Shirakiopsis elliptica | restored | 8.5 |
|  | Albizia grandibracteata | restored | 6.3 |
|  | Rest of the taxa | restored | 43.7 |
|  | Rinorea sp. | primary | 29.7 |
|  | Monodora myristica | primary | 18.9 |
|  | Uvariopsis congensis | primary | 12.2 |
|  | Vepris nobilis | primary | 4.2 |
|  | Lepisanthes senegalensis | primary | 3.8 |
|  | Rest of the taxa | primary | 31.2 |


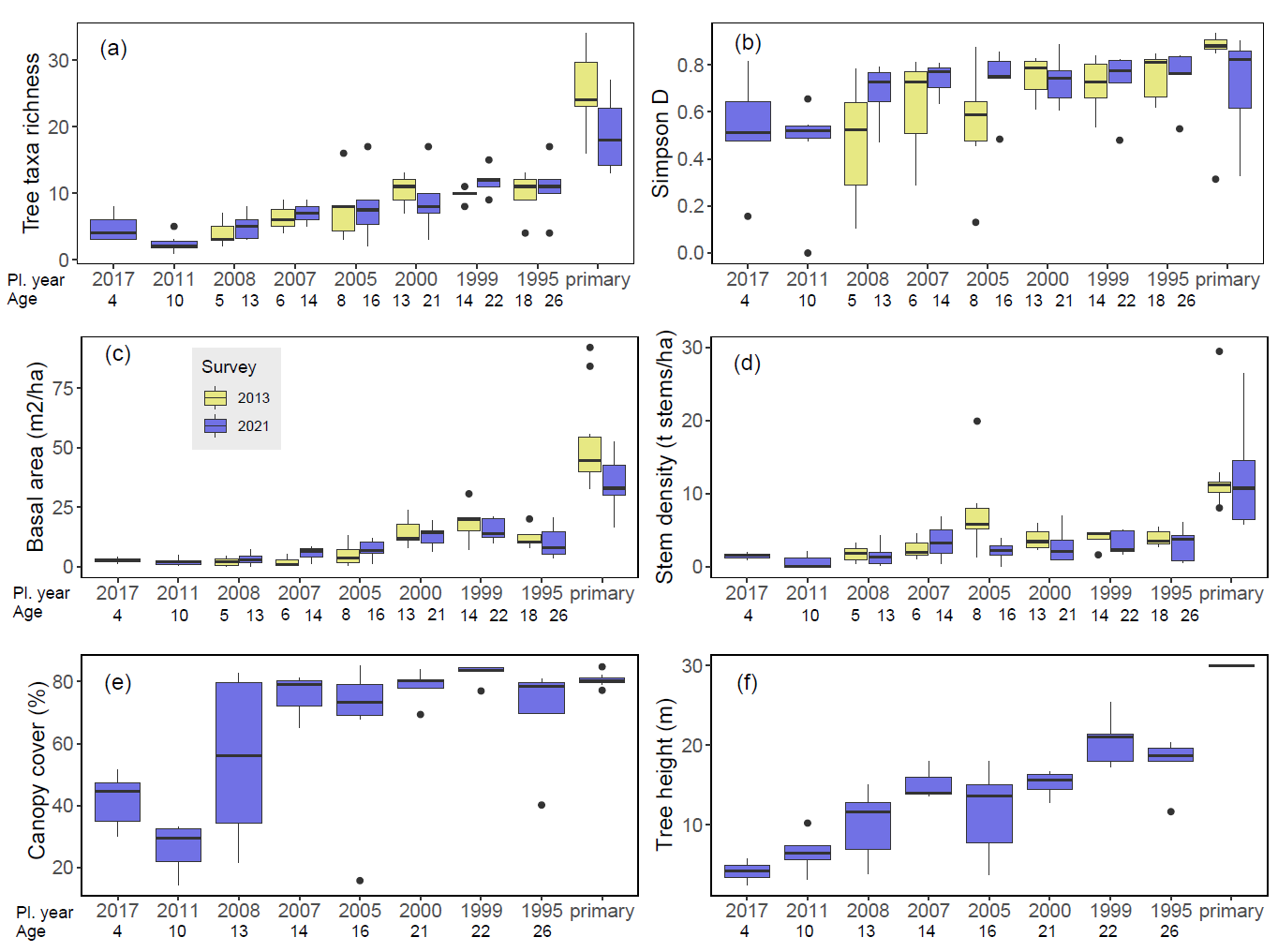


Figure S3. Box plots of variables describing the forest structure in the restoration gradient and primary forest in 2013 and 2021. The x-axis gives both the planting year of the restored sites and the age at the time of the survey. Tree taxa richness per plot (a) and Simpson diversity index (b) per plot. The total estimated basal area (m^2^/ha) (c) and the total estimated stem density (thousand stems/ha) (d). Mean canopy cover (%) per plot (e) and mean tree height (m) per plot of planted trees for restored forests and approximate mean tree height of primary forest in Kibale based on Wing & Buss (1970) (f). Boxes present median, interquartile range, and default whiskers; circles represent outliers (R Core Team, 2023).


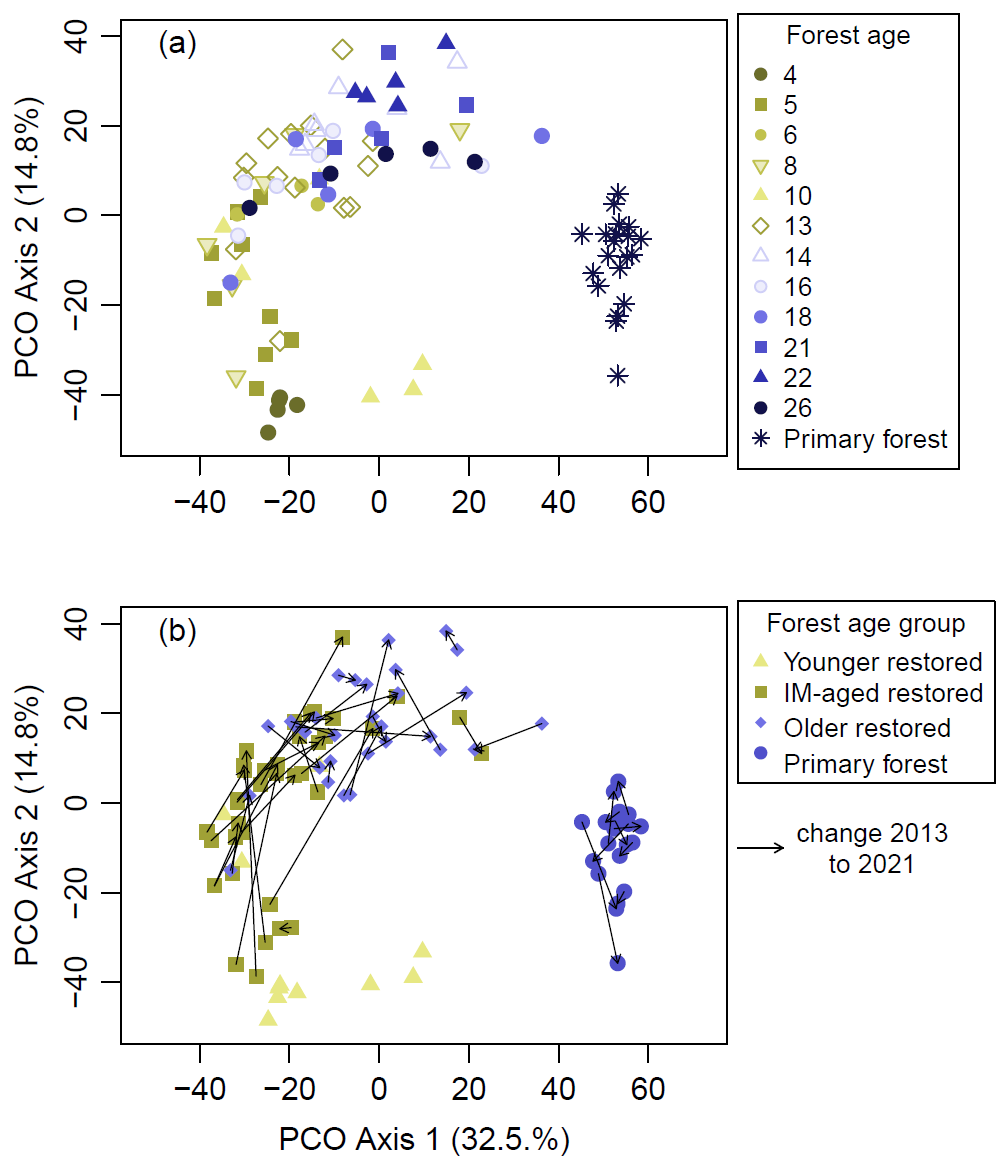


Figure S4. PCO ordination of tree communities in restoration area and primary forests (presence/absence transformation and Sørensen similarity index). The symbols show the forest age during the survey (a) and the change in the tree community compositions of each study site from 2013 to 2021 in the four forest age groups (b). Percentages on the axes show how much the axis explains the total variation. Forest age groups: younger restored (aged 4-10 years in 2021), IM-aged = intermediate-aged restored (aged 5–8 years in 2013, 13–16 years in 2021), older restored (aged 13–18 years in 2013, 21–26 years in 2021), and PF = primary forest.


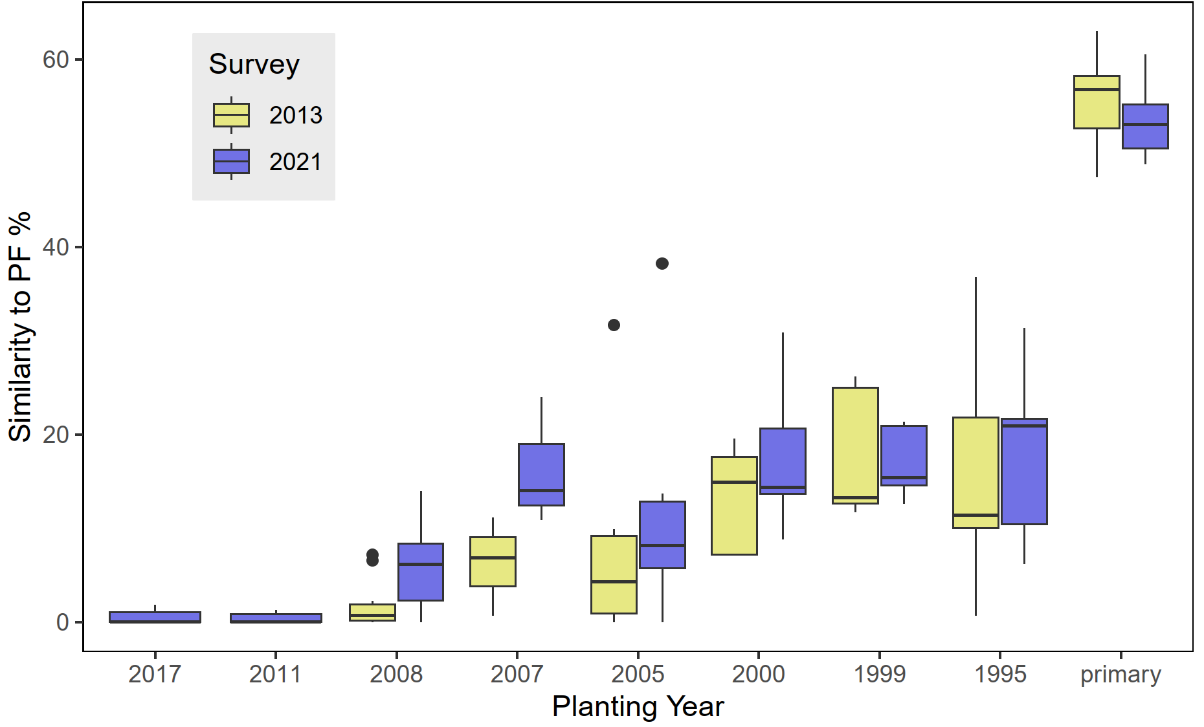


Figure S5. Grouped boxplots presenting Sørensen similarity index (%) to primary forest study sites in 2013 and 2021. The figure shows also the community similarity among the primary forest study sites. Boxes present median, interquartile range, and default whiskers; circles represent outliers (R Core Team, 2023).


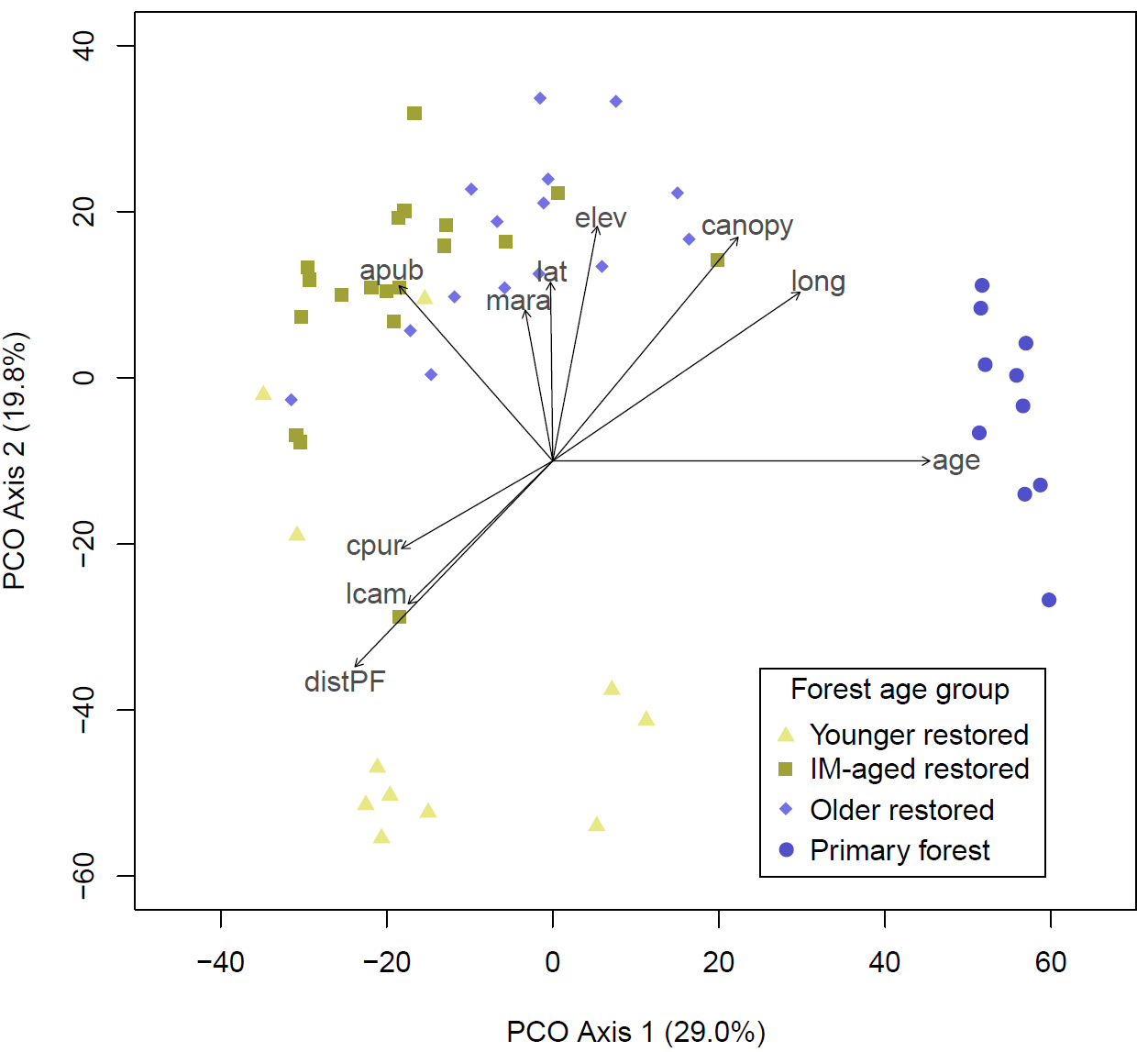


Figure S6. PCO ordination of tree communities in restoration area and primary forests in 2021. Arrows are Pearson correlations of environmental variables. Abbreviations: forest age (age), latitude (lat) and longitude (long), elevation (elev), distance to primary forest (distPF), canopy cover (canopy), vegetation cover estimations of *Acanthus pubescens* (apub), *Lantana camara* (lcam), *Marantochloa* spp. (mara), and *Cenchurus purpureus* (cpur). IM-aged = intermediate-aged.

Table S3. Results of distance-based linear models (DistLM) for tree communities. Models were fitted separately to the two survey times. Prop. = proportion of the variation explained by a variable.

| **MARGINAL TESTS** | |  |  |  |
| --- | --- | --- | --- | --- |
| **Variable** | **SS (trace)** | **Pseudo-F** | **P** | **Prop.** |
| ***2013*** |  |  |  |  |
| Age | 42604.0 | 23.44 | <0.001 | 0.358 |
| Distance to PF | 16817.0 | 6.92 | <0.001 | 0.141 |
| Elevation | 9794.7 | 3.77 | 0.008 | 0.082 |
| Latitude | 7044.0 | 2.64 | 0.029 | 0.059 |
| Longitude | 25990.0 | 11.75 | <0.001 | 0.219 |
| res.df: 42 |  |  |  |  |
| ***2021*** |  |  |  |  |
| Age | 39540.0 | 17.46 | <0.001 | 0.248 |
| Distance to PF | 21175.0 | 8.11 | <0.001 | 0.133 |
| Elevation | 14050.0 | 5.12 | <0.001 | 0.088 |
| Latitude | 8523.3 | 2.99 | 0.006 | 0.053 |
| Longitude | 24985.0 | 9.84 | <0.001 | 0.157 |
| res.df: 53 |  |  |  |  |

Table S4. Results of distance-based linear models (DistLM) for unplanted (naturally germinated) tree communities. Models were fitted separately to the two survey times. Prop. = proportion of the variation explained by a variable.

| **MARGINAL TESTS** | |  |  |  |
| --- | --- | --- | --- | --- |
| **Variable** | **SS(trace)** | **Pseudo-F** | **P** | **Prop.** |
| ***2013*** |  |  |  |  |
| Age | 44750 | 27.06 | <0.001 | 0.422 |
| Distance to PF | 18770 | 7.97 | <0.001 | 0.177 |
| Elevation | 7379.6 | 2.77 | 0.030 | 0.070 |
| Latitude | 4806.1 | 1.76 | 0.119 | 0.045 |
| Longitude | 30480 | 14.94 | 0.000 | 0.288 |
| res.df: 37 |  |  |  |  |
| ***2021*** |  |  |  |  |
| Age | 34206 | 12.54 | <0.001 | 0.194 |
| Distance to PF | 22585 | 7.65 | <0.001 | 0.128 |
| Elevation | 16381 | 5.33 | <0.001 | 0.093 |
| Latitude | 9382.9 | 2.93 | 0.007 | 0.053 |
| Longitude | 25392 | 8.76 | <0.001 | 0.144 |
| res.df: 52 |  |  |  |  |

**References**

Babweteera, F. (2009) ‘Cordia millenii: on the risk of local extinction?’ *African Journal of Ecology*, 47, pp. 367-373.

Barahukwa, A., Chapman, C.A., Namaganda, M., Eilu, G., Omeja, P.A. and Lawes, M.J. (2023) ‘The effects of the invasive species, Lantana camara , on regeneration of an African rainforest’, *African Journal of Ecology*, 61(2), pp. 451–460. Available at: https://doi.org/10.1111/aje.13133.

Duclos, V., Boudreau, S. and Chapman, C.A. (2013) ‘Shrub Cover Influence on Seedling Growth and Survival Following Logging of a Tropical Forest’, *Biotropica*, 45(4), pp. 419–426. Available at: https://doi.org/10.1111/btp.12039.

Duncan, R.S. and Chapman, C.A. (1999) ‘Seed Dispersal and Potential Forest Succession in Abandoned Agriculture in Tropical Africa’, *Ecological Applications*, 9(3), pp. 998–1008. Available at: https://doi.org/10.1890/1051-0761(1999)009[0998:SDAPFS]2.0.CO;2

Forest Monitoring Tools. (2023). *Forest Monitoring Tools*. [Application]. Available at: <https://sites.google.com/view/forestmonitoringtools/home>. (Accessed: 23 December 2024).

Fujita. T. (2021) ‘Fire suppression and seed dispersal play critical roles in the establishment of tropical forest tree species in southeastern Africa’, *Scientific reports*. 11, p. 16391. Available at: https://doi.org/10.1038/s41598-021-95752-7

Hawthorne, W.D. (1995) Ecological Profiles of Ghanaian Forest Trees. Oxford, Oxford Forestry Institute. p. 345.

Hijmans, R.J., Karney, C., Williams, E., and Vennes, C. (2021) ‘Package ‘geosphere’.’ R-project. Available at: https://cran.r-project.org/web/packages/geosphere/geosphere.pdf .

Howard, P., Davenport, T. and Matthews, R. (1996) *Kibale National Park Biodiversity Report*. Kampala: Uganda Forest Department.

IBM Corp. Released 2023. *IBM SPSS Statistics for Windows, Version 29.0.2.0* Armonk, NY: IBM Corp.

Jacob, A.L., Lechowicz, M.J. and Chapman, C.A. (2017) ‘Non‐native fruit trees facilitate colonization of native forest on abandoned farmland’, *Restoration Ecology*, 25(2), pp. 211–219. Available at: <https://doi.org/10.1111/rec.12414>.

Kalema, J. and Hamilton, A. (2020) *Field Guide to the Forest Trees of Uganda. For Identification and Conservation*. Surrey, CABI. p. 277.

Loha, A., Tigabu, M., Teketay, D., Lundkvist, K. and Fries, A. (2006) ‘Provenance Variation in Seed Morphometric Traits, Germination, and Seedling Growth of Cordia africana Lam.’, *New Forest*, 32, pp. 71–86. https://doi.org/10.1007/s11056-005-3872-2

Lusk, C.H. (2022) ‘A field test of forest canopy structure measurements with the CanopyCapture smartphone application’, *PeerJ*, 10, p. e13450. Available at: <https://doi.org/10.7717/peerj.13450>.

Lwanga, J.S. (2003) ‘Forest succession in Kibale National Park, Uganda: implications for forest restoration and management’, *African Journal of Ecology*, 41, pp. 9–22.

Nyafwono, M., Valtonen, A., Nyeko, P., Owiny, A.A., and Roininen, H. (2015) ’Tree community composition and vegetation structure predicted butterfly community recovery in a restored Afrotropical forest’, *Biodiversity and Conservation*, 24, pp- 1473–1485. Available at; https://doi.org/10.1007/s10531-015-0870-3.

Omeja, P.A., Chapman, C.A., Obua, J., Lwanga, J.S., Jacob, A.L., Wanyama, F. and Mugenyi, R. (2011) ‘Intensive tree planting facilitates tropical forest biodiversity and biomass accumulation in Kibale National Park, Uganda’, *Forest Ecology and Management*, 261(3), pp. 703–709. Available at: https://doi.org/10.1016/j.foreco.2010.11.029.

Owiny, A.A., Valtonen, A., Nyeko, P., Malinga, G.M. and Roininen, H. (2016). ’Tree communities of different aged logged areas in an Afrotropical rainforest’, *African Journal of Ecology*, 54: 207—2016.

Patel, N. (2018) *CanopyCapture*. [Application]. Available at: <https://nikp29.github.io/CanopyCapture/> (Accessed: 23 January 2023).

R Core Team. (2023*) R: A Language and Environment for Statistical Computing*. Vienna: R Foundation for Statistical Computing. Available at <https://www.r-project.org/>.

Schleuning, M., Blüthgen, N, Flörchinger, M., Braun, J., Schaefer, H.M. and Böhning-Gaese, K. (2011) ‘ Specialization and interaction strength in a tropical plant-frugivore network differ among forest strata’, *Ecology*, 92(1), pp. 26-36.

Sheil, D., Jennings, S. and Savill, P. (2000) ‘Long-term permanent plot observations of vegetation dynamics in Budongo, a Ugandan rain forest’, *Journal of Tropical Ecology*, 16, pp. 765–800.

Sheil, D., Salim, A., Chave, J., Vanclay, J.K. and Hawthorne, W.D. (2006) Illumination-size relationships of 109 coexisting tropical forest trees’, *Journal of Ecology*, 94: pp. 494–507.

Ssekuubwa, E., van Goor, W., Snoep, M., Riemer, K., Wanyama, F. and Tweheyo, M. (2021) ‘Recovery of seedling community attributes during passive restoration of a tropical moist forest in Uganda’, *Applied Vegetation Science*, 24, e12559. Available at: <https://doi.org/10.1111/avsc.12559> .

Tabuti, J.R.S. (2007) ‘The uses, local perceptions and ecological status of 16 woody species of Gadumire Sub-county, Uganda’, *Biodiversity and Conservation*, 16, pp. 1901–1915. Available at: https://doi.org/10.1007/s10531-006-9097-7.

UWA-FACE. (2015) *CCB Project Implementation Report*. Uganda Wildlife Authority, Face the Future, p. 14. Available at: <https://registry.verra.org/app/projectDetail/VCS/673> (Accessed: 13 September 2024).

UWA-FACE. (2017) *CCB Project Implementation Report 2014-2017*. Uganda Wildlife Authority, Face the Future, p. 16. Available at: <https://registry.verra.org/app/projectDetail/VCS/673> (Accessed: 13 September 2024).

Valtonen, A.,Korkiatupa, E., Holm, S., Malinga, G.M., Nakadai, and R. (2021) ‘Remotely sensed vegetation greening along a restoration gradient of a tropical forest, Kibale National Park, Uganda’, *Land degradation and Development*, 32, pp. 5166–5177. Available at: https://doi.org/10.1002/ldr.4096 .

van Goor, W. (2021) CCB MONITORING REPORT 2017 – 2020. Face the Future, Uganda Wildlife Authority, p. 47. Available at: <https://registry.verra.org/app/projectDetail/VCS/673> (Accessed: 13 September 2024).

Wheeler, C.E., Omeja, P.A., Chapman, C.A., Glipin, M., Tumwesigye, C. and Lewis, S.L. (2016) ‘Carbon sequestration and biodiversity following 18 years of active tropical forest restoration’, *Forest Ecology and Management*, 373, pp. 44–55. Available at: https://doi.org/10.1016/j.foreco.2016.04.025.

Wing, L.D. and Buss, I.O. (1970) ‘Elephants and Forests’, *Wildlife Monographs*, (19), pp. 3–92.

Zanne, A.E., Chapman, C.A. and Kitajima, K. (2005). ‘Evolutionary and ecological correlates of early seedling morphology in East African trees and shrubs’, *American Journal of Botany*, 92, p. 972-978.
