# Supplementary material for: Chronosequence Resampling Elucidates Tree Community and Forest Structure Recovery Patterns in Restored Tropical Rainforest
Source: Ecol Evol. 2025 Aug 24;15(8):e72033. doi: 10.1002/ece3.72033 (PMC12375826; doi:10.1002/ece3.72033)
Supplement: Supplementary file 2 — Appendix S2. [file ECE3-15-e72033-s001.docx]

Ecology & Evolution

Appendix S2

**Title:** Chronosequence resampling elucidates tree community and forest structure recovery patterns in restored tropical rainforest

**Authors:** Eveliina Korkiatupa, Geoffrey M. Malinga, Sille Holm, Wouter van Goor, Richard Kigenyi, Anu Valtonen

**Understorey vegetation covers**

*Materials and methods*

In the naturally regenerating forest gaps of Kibale National Park, the grass, shrub and herb cover follows successional phases described by Wing and Buss (1970); the decline of the grass cover and the increase of the shrub and herb cover coincides with the closing canopy. We wanted to know if similar phases can be found also in the actively restored areas in the cover of the most dominant grass, shrub and herb species. These include elephant grass (*Cenchurus purpureus*), native shrub *Acanthus pubescens*, tall-growing herbs belonging to the genus *Marantochloa*, and common lantana (*Lantana camara*), which is an invasive shrub in Kibale. We chose *C. purpureus*, A*. pubescens* and *L. camara*, because when they are very abundant, they can hinder tree recruitment (Duncan & Chapman, 1999; Omeja et al., 2011; Duclos, Boudreau and Chapman, 2013; Barahukwa et al. 2023). We expect these species’ covers will follow the same successional changes as observed in the naturally regenerating forest gaps, i.e., the decline of elephant grass followed by a temporary increase in *A. pubescens* and *L. camara* in the intermediate age classes, followed by the shade tolerant herbs *Marantochloa* spp. (Wing and Buss 1970).

We visually assessed cover estimates (0-100%; rounded to the nearest 5 %) of (7) a dominant grass *C*. *purpureus*, (8) a woody shrub *A*. *pubescens*, (9) an invasive shrub *L*. *camara*, and (10) herbs belonging to *Marantochloa* spp. To visualise the changes in the understorey vegetation, we also plotted the vegetation covers (%) of *C*. *purpureus*, *A*. *pubescens*, *L*. *camara*, and *Marantochloa* spp. across the forest age gradient. We also visualised the growth rate of the planted trees by plotting the DBH (cm) of each planted tree against the age since planting and then fitted linear regression models separately for each species and for both survey times, using R (R Core Team 2023).

*Results and discussion*

The vegetation cover estimates showed mainly non-linear patterns along the restoration age gradient (Appendix S2: Fig. S1). The elephant grass *C. purpureus* cover reached maximum 4–13 years after the restoration planting but was practically absent in older restored forests. The native shrub *A. pubescens* cover reached its highest levels approximately 13 years after planting. The invasive species *L. camara* cover varied between 0% and 25% in younger and intermediate-aged restored forests. The native shrub *Marantochloa* spp. cover was greatest in older restored forests and low in the primary forest reference sites.


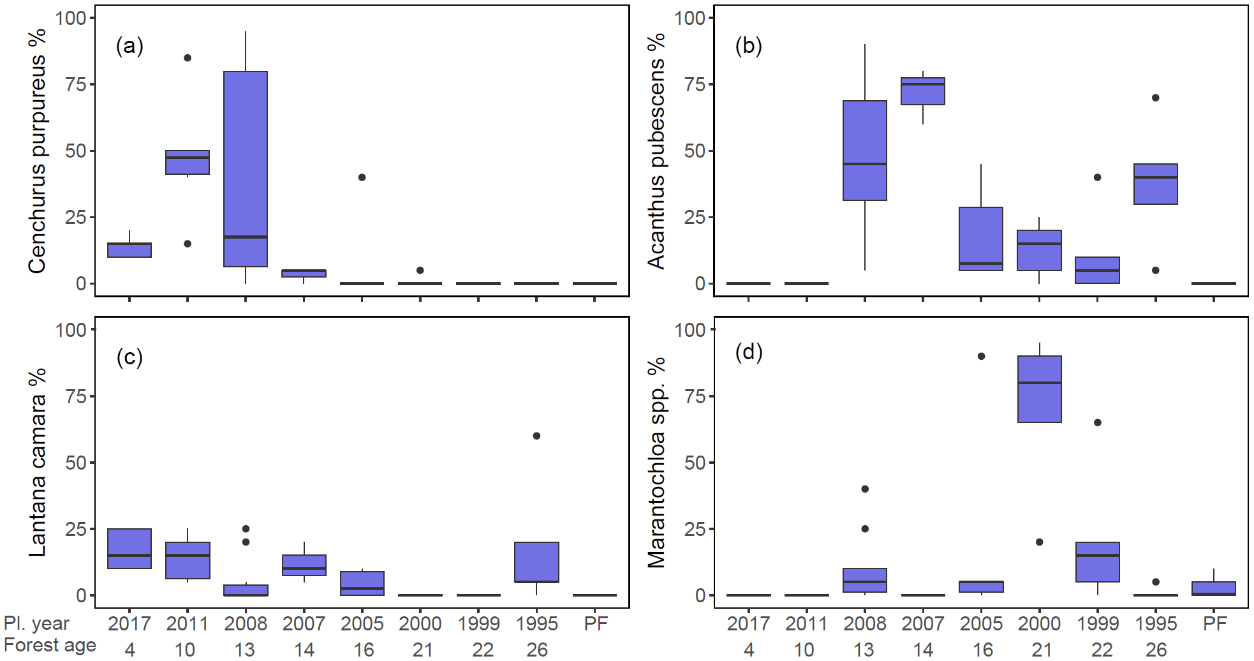


Figure S1. The vegetation cover estimates (%) of *Cenchurus purpureus* (a), *Acanthus pubescens* (b), *Lantana camara* (c), and *Marantochloa* spp. (d) in the 2021 survey. Boxes present median, interquartile range, and default whiskers; circles represent outliers (R Core Team, 2023).

Patterns in covers of *C.* *purpureus*, *A.* *pubescens*, *L*. *camara* and *Marantochloa* spp. followed the corresponding patterns reported from successional stages in forest gaps within primary forests of Kibale (Wing and Buss 1970). Especially *C. purpureus* and *A. pubescens* have been shown to limit tree establishment in Kibale (Duncan and Chapman, 1999; Omeja et al., 2011; Duclos, Boudreau and Chapman, 2013), but we found that their cover shrunk generally towards older restored forests. This result implies that the established planted trees and new recruits (result of active restoration) have shaded out these aggressive grass and shrub species. Amplification of shade-tolerant *Marantochloa* spp. could suggest that transition from stand initiation to stem exclusion phase has started (also seen in the Appendix S1: Fig. S6). Estimating dominant species cover of understorey vegetation can give additional information for monitoring the recovery process in tropical forest restoration areas. For example, cover values could help identify possible challenges in the recovery process or help to mechanistically understand arrested succession, if that should occur.

Invasive or aggressive species can hinder ecosystem recovery in the restoration (Cordell et al., 2016; Weidlich et al., 2020). In our restored study sites, the cover of a Neotropical shrub *L. camara* varied between 0% and 60%. *L*. *camara* is a harmful invasive species across the globe (Goncalves et al., 2014). Consequently, the UWA-FACE project has been actively removing *L*. *camara* and other exotic species from the restoration area (van Goor, 2021).

**Tree growth rate**

*Materials and methods*

Planted trees form an essential element of restored forest structure at the beginning of succession. We were interested to know which planted species have established themselves, and their annual diameter growth rate. The species composition of planted trees was designed to contain species with high survival rates in Kibale (Wheeler et al., 2016), so we expected that the planted trees would grow annually, producing an increase of DBH along the chronosequence age gradient.

We chose only species with ≥ 10 individuals across the restoration age gradient. In these analyses, 2013 survey data (information on which tree was planted vs. non-planted) was missing from five study sites that were planted in 1995 (N = 1/5 study sites), 1999 (N = 2/5), and 2000 (N = 2/5).

To determine the rate at which the planted trees are growing in the restored areas, we plotted the DBH (cm) of each planted tree against the forest age (since planting) and fitted linear regression models separately for both survey times in R Studio.

*Results*

From 89 tree taxa recorded in the surveys, seven were also found as planted: *Bridelia micrantha*, *Croton* sp., *Maesa lanceolata*, *P*runus *africana*, *Shirakiopsis elliptica*, *Spathodea campanulata*, and *Warburgia ugandensis*. Only four species had enough DBH measures to run linear models. The growth rate of DBH for *B. micrantha* was almost 1 cm/yr (Appendix S2; Fig. S2a). For *S. elliptica*, the linear model for 2013 data estimated a growth rate of 1.6 cm/yr, and model for 2021 data, a growth rate of 0.9 cm/yr (Appendix S2: Fig. S2b). Linear models were nonsignificant for *S. campanulata* or *W. ugandensis* (Appendix S2: Fig. S2c,d).


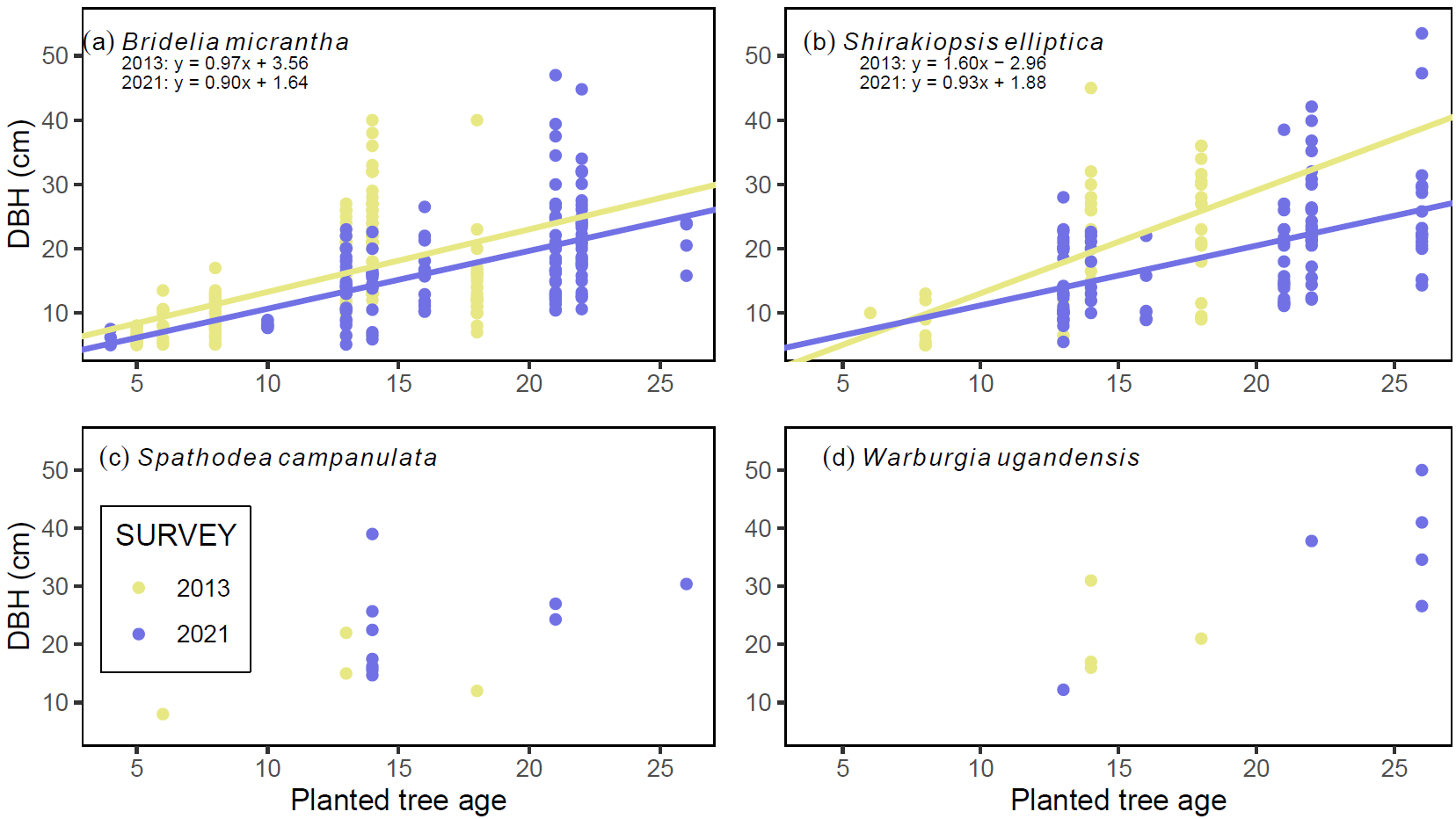


Figure S2. DBH (cm) of the four planted species (a-d) with more than 10 observations plotted against the tree age. Linear models were fitted separately for each survey time and are shown only if statistically significant (p < 0.05; R Core Team, 2023). Results of linear models for *B. micrantha* 2013: R^2^ = 0.282, F (1,154) = 60.52, p < 0.001, and 2021: R^2^ = 0.297, F (1,123) = 51.92, p < 0.001. Results of linear models for *S. elliptica* 2013: R^2^ = 0.336, F (1,39) = 19.73, p < 0.001, and 2021: R^2^ = 0.255, F (1,100) = 34.17, p < 0.001.

**References**

Cordell, S., Ostertag, R., Michaud, J. and Warman, L. (2016) ‘Quandaries of a decade‐long restoration experiment trying to reduce invasive species: beat them, join them, give up, or start over?’, *Restoration Ecology*, 24(2), pp. 139–144. Available at: <https://doi.org/10.1111/rec.12321>.

Barahukwa, A., Chapman, C.A., Namaganda, M., Eilu, G., Omeja, P.A. and Lawes, M.J. (2023) ‘The effects of the invasive species, Lantana camara , on regeneration of an African rainforest’, African Journal of Ecology, 61(2), pp. 451–460. Available at: <https://doi.org/10.1111/aje.13133>.

Cordell, S., Ostertag, R., Michaud, J. and Warman, L. (2016) ‘Quandaries of a decade‐long restoration experiment trying to reduce invasive species: beat them, join them, give up, or start over?’, *Restoration Ecology*, 24(2), pp. 139–144. Available at: <https://doi.org/10.1111/rec.12321>.

Duclos, V., Boudreau, S. and Chapman, C.A. (2013) ‘Shrub Cover Influence on Seedling Growth and Survival Following Logging of a Tropical Forest’, Biotropica, 45(4), pp. 419–426. Available at: <https://doi.org/10.1111/btp.12039>.

Duncan, R.S. and Chapman, C.A. (1999) ‘Seed Dispersal and Potential Forest Succession in Abandoned Agriculture in Tropical Africa’, Ecological Applications, 9(3), pp. 998–1008. Available at: [https://doi.org/10.1890/1051-0761(1999)009[0998:SDAPFS]2.0.CO;2](https://doi.org/10.1890/1051-0761(1999)009%5b0998:SDAPFS%5d2.0.CO;2).

Goncalves, E., Herrera, I., Duarte, M., Bustamante, R.O., Lampo, M., Velásquez, G., Sharma, G.P. and García-Rangel, S. (2014) ‘Global Invasion of Lantana camara: Has the Climatic Niche Been Conserved across Continents?’, *PLoS ONE*, 9(10), p. e111468. Available at: <https://doi.org/10.1371/journal.pone.0111468>.

Omeja, P.A., Chapman, C.A., Obua, J., Lwanga, J.S., Jacob, A.L., Wanyama, F. and Mugenyi, R. (2011) ‘Intensive tree planting facilitates tropical forest biodiversity and biomass accumulation in Kibale National Park, Uganda’, Forest Ecology and Management, 261(3), pp. 703–709. Available at: <https://doi.org/10.1016/j.foreco.2010.11.029>.

R Core Team. (2023) *R: A Language and Environment for Statistical Computing*. Vienna: R Foundation for Statistical Computing. Available at <https://www.r-project.org/>.

van Goor, W. (2021) *CCB MONITORING REPORT 2017 – 2020*. Face the Future, Uganda Wildlife Authority, p. 47. Available at: <https://registry.verra.org/app/projectDetail/VCS/673> (Accessed: 13 September 2024).

Weidlich, E.W.A., Flórido, F.G., Sorrini, T.B. and Brancalion, P.H.S. (2020) ‘Controlling invasive plant species in ecological restoration: A global review’, *Journal of Applied Ecology*, 57(9), pp. 1806–1817. Available at: <https://doi.org/10.1111/1365-2664.13656>.

Wheeler, C.E., Omeja, P.A., Chapman, C.A., Glipin, M., Tumwesigye, C. and Lewis, S.L. (2016) ‘Carbon sequestration and biodiversity following 18 years of active tropical forest restoration’, *Forest Ecology and Management*, 373, pp. 44–55. Available at: <https://doi.org/10.1016/j.foreco.2016.04.025>.

Wing, L.D. and Buss, I.O. (1970) ‘Elephants and Forests’, *Wildlife Monographs*, (19), pp. 3–92.
